# ClinOmicsTrail^bc^: a visual analytics tool for breast cancer treatment stratification

**DOI:** 10.1093/bioinformatics/btz302

**Published:** 2019-04-30

**Authors:** Lara Schneider, Tim Kehl, Kristina Thedinga, Nadja Liddy Grammes, Christina Backes, Christopher Mohr, Benjamin Schubert, Kerstin Lenhof, Nico Gerstner, Andreas Daniel Hartkopf, Markus Wallwiener, Oliver Kohlbacher, Andreas Keller, Eckart Meese, Norbert Graf, Hans-Peter Lenhof

**Affiliations:** 1 Center for Bioinformatics, Saarbrücken, Germany; 2 Saarbrücken Graduate School of Computer Science, Saarbrücken, Germany; 3 Chair for Clinical Bioinformatics, Saarland Informatics Campus, Saarland University, Saarbrücken, Germany; 4 Quantitative Biology Center (QBiC), Tübingen, Germany; 5 Institute for Translational Bioinformatics, University Hospital Tübingen, Tübingen, Germany; 6 Department of Systems Biology, Boston, MA, USA; 7 Department of Cell Biology, Harvard Medical School, Boston, MA, USA; 8 cBio Center, Dana-Farber Cancer Institute, Boston, MA, USA; 9 Department of Obstetrics and Gynecology, University of Tübingen, Tübingen, Germany; 10 Department of Obstetrics and Gynecology, University of Heidelberg, Heidelberg, Germany; 11 National Center for Tumor Diseases, University of Heidelberg, Heidelberg, Germany; 12 Center for Bioinformatics, University of Tübingen, Tübingen, Germany; 13 Applied Bioinformatics, Department of Computer Science, University of Tübingen, Tübingen, Germany; 14 Biomolecular Interactions, Max Planck Institute for Developmental Biology, Tübingen, Germany; 15 Human Genetics, Saarland University, Homburg, Germany; 16 Department of Pediatric Oncology and Hematology, Medical School, Saarland University, Homburg, Germany

## Abstract

**Motivation:**

Breast cancer is the second leading cause of cancer death among women. Tumors, even of the same histopathological subtype, exhibit a high genotypic diversity that impedes therapy stratification and that hence must be accounted for in the treatment decision-making process.

**Results:**

Here, we present ClinOmicsTrail^bc^, a comprehensive visual analytics tool for breast cancer decision support that provides a holistic assessment of standard-of-care targeted drugs, candidates for drug repositioning and immunotherapeutic approaches. To this end, our tool analyzes and visualizes clinical markers and (epi-)genomics and transcriptomics datasets to identify and evaluate the tumor’s main driver mutations, the tumor mutational burden, activity patterns of core cancer-relevant pathways, drug-specific biomarkers, the status of molecular drug targets and pharmacogenomic influences. In order to demonstrate ClinOmicsTrail^bc^’s rich functionality, we present three case studies highlighting various ways in which ClinOmicsTrail^bc^ can support breast cancer precision medicine. ClinOmicsTrail^bc^ is a powerful integrated visual analytics tool for breast cancer research in general and for therapy stratification in particular, assisting oncologists to find the best possible treatment options for their breast cancer patients based on actionable, evidence-based results.

**Availability and implementation:**

ClinOmicsTrail^bc^ can be freely accessed at https://clinomicstrail.bioinf.uni-sb.de.

**Supplementary information:**

Supplementary data are available at *Bioinformatics* online.

## 1 Introduction

Breast cancer is the most common type of cancer among women (Siegel *et al.*, 2017). As current standard of care, treatment options are typically selected based on a few clinical markers like cancer stage, the presence or absence of hormone receptors, HER2/neu amplification and the menopausal status of the patient (https://www.cancer.org/cancer/breast-cancer/treatment/treatment-of-breast-cancer-by-stage.html). Several tools and web services have been developed to support the breast cancer treatment decision-making process. Some of them solely analyze a rather small set of clinical markers to estimate treatment benefits for various hormonal and chemotherapeutic agents (http://www.lifemath.net/cancer/breastcancer/therapy/, dos Reis *et al.*, 2017). Other approaches focus on medium-sized prognostic gene expression assays that assess the risk for relapse and metastasis (e.g. Oncotype DX or MammaPrint). Based on these predictions, the benefit of adjuvant chemotherapy can be determined. However, these tests do not consider the effect of mutations that might drive the disease or cause resistance to certain drugs.

Accounting for the influence of mutations on treatment response, we recently proposed DrugTargetInspector (Schneider *et al.*, 2016), a general purpose assistance tool for the investigation of differentially expressed or mutated molecular targets of known drugs, as well as their involvement in dysregulated signaling pathways.

Here, we present ClinOmicsTrail^bc^, an interactive visual analytics tool for breast cancer treatment stratification. ClinOmicsTrail^bc^ supports oncologists in the breast cancer treatment decision-making process by providing a thorough assessment of a variety of treatment options. Our tool comprehensively surveys various classes of standard-of-care targeted drugs including aromatase inhibitors, estrogen receptor-targeting drugs, angiogenesis inhibitors, as well as drugs targeting ERBB2, MTOR, CDK, or PARP. Additionally, ClinOmicsTrail^bc^ evaluates immunotherapeutic approaches including checkpoint inhibitors and personalized cancer vaccines as putative treatment options. Moreover, the web service examines driver-targeting drugs that are approved for other cancer types and that hence can be considered as candidates for drug repositioning. To serve as a firm basis for these suggestions, ClinOmicsTrail^bc^ conducts a multitude of analyses on the tumor’s clinical markers and (epi-)genomic and transcriptomic alterations to determine the tumor’s main driver mutations and its mutational burden, to identify activity patterns of cancer-relevant pathways, and to investigate the status of drug-specific biomarkers, molecular drug targets, involved drug-processing enzymes and efflux transporters. The identified characteristics are then summarized in easy-to-interpret interactive visualizations that provide clinicians with treatment-relevant and actionable information at the point of care.

Compared to the approaches mentioned above, ClinOmicsTrail^bc^ excels in breadth and depth of the provided functionality (see Supplementary File S1).

## 2 Materials and methods

ClinOmicsTrail^bc^ guides breast cancer therapy selection by evaluating available therapeutic regimens in the context of the (epi-)genetic and molecular tumor characteristics. To this end, ClinOmicsTrail^bc^ analyzes and integrates clinical, (epi-)genomics and transcriptomics data with a priori knowledge from clinical practice guidelines and various medical, pharmacological and biological databases to assess therapy options, both on-label and off-label. ClinOmicsTrail^bc^ reports these results in an intuitive and interactive manner, highlighting characteristics that might support or contraindicate the use of specific therapy options.

**Fig. 1.**
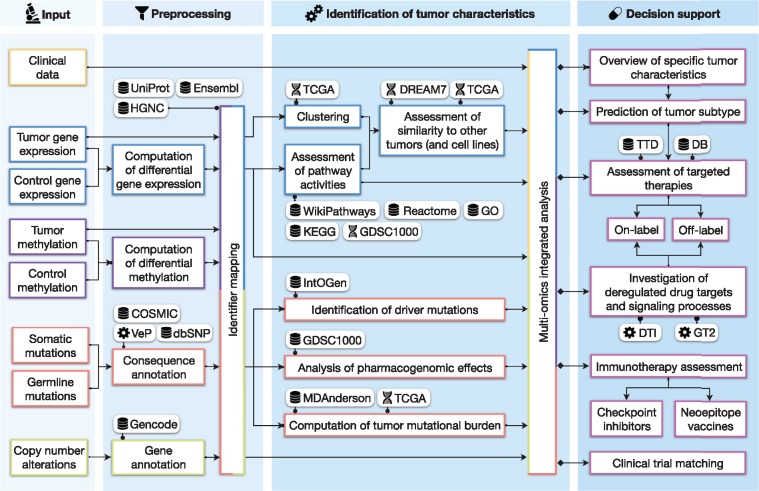
Overview of ClinOmicsTrail^bc^ workflow. Integrated databases are indicated by a database icon, third party tools by a gear wheel and molecular datasets by the double-helix symbol. COSMIC: Catalogue Of Somatic Mutations In Cancer, DB: DrugBank, DREAM7: Dialogue for Reverse Engineering Assessments and Methods—drug sensitivity prediction challenge, DTI: DrugTargetInspector, GDSC1000: Genomics of Drug Sensitivity in Cancer, GO: Gene Ontology, GT2: GeneTrail2, HGNC: HUGO Gene Nomenclature Committee, IntOGen: Integrative Onco Genomics, KEGG: Kyoto Encyclopedia of Genes and Genomes, MDAnderson: MD Anderson Cancer Center, TCGA: The Cancer Genome Atlas, TTD: Therapeutic Target Database, VeP: Variant Effect Predictor

Figure 1 gives an overview of the ClinOmicsTrail^bc^ workflow, covering all steps from sample data input, over preprocessing and analysis steps, to the interactive visualization of the results. The respective workflow components will be described in more detail in the following sections. Section 2.1 provides details on the considered types of input data and their relevance for the treatment decision-making process. Various types of analyses for the identification of specific tumor characteristics are presented in Section 2.2. Finally, Section 2.3 illustrates how these characteristics are used to inform treatment stratification. For details on the implementation of the web service, please refer to Supplementary File S2.

### 2.1 Input data and preprocessing

ClinOmicsTrail^bc^ allows the user to integrate clinical data of the tumor under investigation with corresponding (epi-)genetic alterations and gene expression measurements (cf. Fig. 1, first and second column).

To this end, our web service analyzes the status of several standard clinical markers for breast cancer diagnosis and treatment: presence of hormone receptors, menopausal status of the patient, amplification of the human epidermal growth factor receptor 2 (HER2/neu). Also, information on tumor growth (Ki-67 staining, S-phase fraction), the histopathological subtype, tumor size and grade, lymph node and metastasis status, as well as clinical metadata like a patient ID, the origin of the sample (primary tumor versus metastasis), the fraction of tumor tissue in the sample and the date of biopsy can be provided to ClinOmicsTrail^bc^.

Besides the clinical biomarkers, various types of molecular and genetic data can be uploaded and investigated. Based on gene expression measurements of a tumor sample and a matched control (or other tumor samples), ClinOmicsTrail^bc^ computes for each gene either a log-fold-change or a z-score mirroring its differential gene expression. Alternatively, precomputed scores of differential gene expression can also directly be uploaded. These scores are used—among others—to estimate the activity of cancer-relevant signaling pathways (cf. Section 2.2.2). While the comparison of a tumor sample to a healthy control reveals which pathogenic processes are up- or downregulated, the comparison of tumor versus tumor provides a more fine-grained view on the specific characteristics of the sample of interest in comparison to other tumors of the same type or subtype.

For the analysis of genetic variations, ClinOmicsTrail^bc^ requires (whole genome or exome) mutation data of the tumor sample and a control to identify somatic and germline mutations. These will be used for the identification and prioritization of driver mutations, the assessment of pharmacogenomic effects and the computation of tumor mutational burden (cf. Section 2.2.1). Mutation data can be uploaded to ClinOmicsTrail^bc^ in variant call format (.vcf). In order to assess the impact of protein-coding mutations on the disease phenotype, all mutations are annotated with their effects on the corresponding proteins (e.g. missense variant, stop gain, frameshift) using Ensembl’s Variant Effect Predictor (VeP) (McLaren *et al.*, 2016). Additionally, the contained mutations are cross-referenced with dbSNP and COSMIC for further details on the potential functional impact and pathogenicity of the mutation.

As altered copy numbers are believed to account for up to 85% of dysregulated gene expression in breast cancers (Srihari *et al.*, 2016), ClinOmicsTrail^bc^ also considers this type of data for the identification of driver genes and the holistic assessment of altered processes in the tumor. Somatic and germline copy number alterations can be uploaded in segmented data file format (.seg) as log-ratios of tumor copy numbers in relation to normal copy number levels. The copy number alterations of the contained genomic regions are mapped to genes using the reference genomes GRCh37/38 and gene annotations from Gencode (Harrow *et al.*, 2012).

Besides the aforementioned genomic aberrations, also epigenomic changes (e.g. differential methylation of promotors) can contribute to tumor initiation and progression. Methylation data can be provided to ClinOmicsTrail^bc^ as white-space separated file that contains methylation scores (e.g. beta values of promoter methylation) per gene identifier or precomputed scores of differential methylation. In cases where beta values for one tumor sample and one (or several) control samples are provided, ClinOmicsTrail^bc^ computes log-fold-quotients (or z-scores) to assess differential methylation.

For all omics data types, we support all commonly used identifier types, including HGNC gene symbols, NCBI EntrezGene IDs, Ensembl IDs and UniProt identifiers. The identifiers used in the provided omics datasets are unified in an automatic identifier mapping step for a seamless integration in the following analyses.

### 2.2 Identification of specific tumor characteristics

Based on the various types of input data described in Section 2.1, ClinOmicsTrail^bc^ performs a variety of analyses to reveal specific tumor characteristics (cf. Fig. 1, third column). In a first step, ClinOmicsTrail^bc^ identifies tumor-driving (epi-)genomic aberrations including mutations, copy number variations and DNA methylation (Section 2.2.1). As these alterations also manifest in the activities of signaling cascades, we compute pathway activities for a set of 20 core cancer-associated pathways based on the differential gene expression of the involved genes (Section 2.2.2). Finally, based on the complete expression profile, ClinOmicsTrail^bc^ provides a clustering with respect to more than 500 breast cancer profiles from TCGA that allows to assess the tumor’s intrinsic subtype (Section 2.2.3).

#### 2.2.1 Assessment of tumor-driving (epi-)genomic aberrations

Tumors can potentially contain a plethora of mutations that are usually divided into driver and passenger mutations based on their impact on disease development (Haber and Settleman, 2007). For the identification and prioritization of tumor-specific driver genes, ClinOmicsTrail^bc^ uses the IntOGen database (Gonzalez-Perez *et al.*, 2013). ClinOmicsTrail^bc^ provides a prioritized list of putative driver genes found in the tumor and indicates their predicted severity. Clicking on the indicator symbol opens additional details on the detected mutation(s).

Mutations can also modulate a tumor’s response to drugs, both, with respect to efficacy and toxicity (Wheeler *et al.*, 2013). In order to account for such pharmacogenomic effects, ClinOmicsTrail^bc^ investigates the genomic and transcriptomic state of relevant drug-processing enzymes and resistance-promoting factors to evaluate the applicability of drugs or the potentially required adaption in dosage for the considered case (cf. Section 2.3.2). Additionally, for a given mutation, ClinOmicsTrail^bc^ displays the putative impact of this mutation on affected drugs as predicted by the Genomics of Drug Sensitivity in Cancer (GDSC1000) database (Iorio *et al.*, 2016). Finally, the entire mutation data is evaluated to derive the tumor mutational burden, a measure that serves as predictive biomarker for immunotherapy (cf. Section 2.3.3).

Besides small-scale genomic aberrations, our tool also determines the impact of copy number alterations and epigenetic modifications on driver genes and drug sensitivity.

#### 2.2.2 Assessment of pathway activities

Tumor mutations can drive the aberrant activity of key cancer-associated pathways (Giancotti, 2014). In order to obtain an overview of altered processes in a breast tumor, we consider pathway activities of a set of 20 core cancer-relevant pathways (Velloso *et al.*, 2017) that can also be utilized to characterize tumor subtypes. Here, the activity of a pathway is approximated by a weighted sum of the deregulation scores of the pathway genes.

In order to assess the significance of the computed pathway activities, empirical *P*-values are computed based on a user-defined number of permutations on the scores of differential expression. The derived *P*-values are adjusted for multiple hypothesis testing using the Benjamini-Hochberg method (Benjamini and Hochberg, 1995). Details on how the pathway gene sets are constructed and on the computation of the pathway activity scores and their *P*-values can be found in Supplementary Files S3 and S4.

**Fig. 2.**
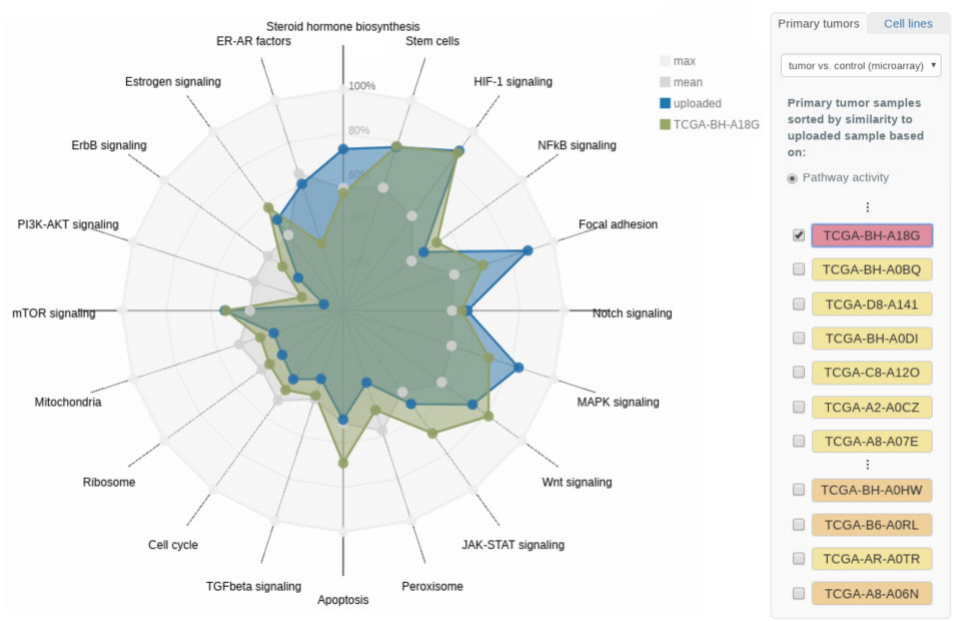
Radar chart of pathway activities. The pathway activities of a set of 20 core cancer-associated pathways for the user-provided tumor sample (TCGA-AN-A0XN, cf. Section 3.1) are shown. Reference samples from TCGA as well as breast cancer cell lines can be added to the visualization interactively. Here, the triple-negative TCGA sample TCGA-BH-A18G shows a similar activity pattern to the sample under investigation. The molecular subtype of the respective reference samples is color-coded in the side panel on the right

The computed pathway activities and their empirical *P*-values are displayed in a radar chart, where each radar axis represents the activity of one of the selected pathways (Fig. 2). As a reference, the pathway activity patterns of more than 500 tumor samples from the TCGA breast cancer cohort and 45 breast cancer cell lines (Costello *et al.*, 2014) can be interactively added to the plot by clicking on the corresponding sample’s checkbox (cf. right panel in Fig. 2). The reference samples are sorted in decreasing order of similarity to the sample under consideration. The similarity is computed as the mean-squared distance of corresponding pathway activity scores. Clicking on the sample name yields information on clinical markers and in the case of the 45 cell lines additionally details on drug sensitivities and growth rates.

#### 2.2.3 Clustering

**Fig. 3.**
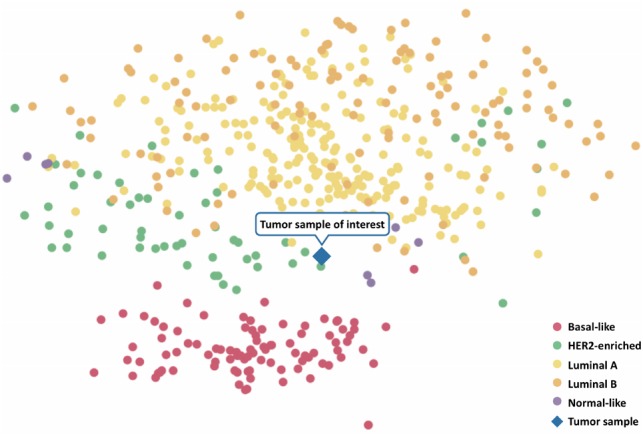
Exemplary clustering results. An uploaded tumor gene expression sample (TCGA-AN-A0XN, cf. Section 3.1) is clustered along with breast tumor samples from TCGA. The molecular subtypes of the TCGA samples are color-coded as indicated by the legend in the lower right corner. The tumor sample under investigation is indicated by the diamond-shaped symbol

There are four main molecular subtypes of breast cancer (luminal A, luminal B, HER2-enriched and basal-like) that differ in the composition of relevant receptors and their respective growth rates, but also in their gene expression patterns (Neve *et al.*, 2006). In order to investigate a sample’s intrinsic subtype, we compute a clustering of the sample under investigation in comparison to more than 500 breast tumor samples obtained from TCGA (Grossman *et al.*, 2016). To this end, we use the classic Principal Component Analysis (PCA) (Wold *et al.*, 1987) as well as t-distributed Stochastic Neighbor Embedding (t-SNE) (Maaten and Hinton, 2008), a non-linear dimension reduction technique that captures the similarity of samples in a two-dimensional space. In the resulting visualization, TCGA’s samples are color-coded according to their molecular subtypes (The Cancer Genome Atlas Network, 2012) (Fig. 3).

### 2.3 Decision support functionality

ClinOmicsTrail^bc^ offers an integrated analysis of the key tumor characteristics (cf. Section 2.2) to support the clinical treatment decision-making process with respect to a variety of treatment options (cf. Fig. 1, fourth column). As a starting point, ClinOmicsTrail^bc^ provides a compact visualization of the tumor’s genomic and transcriptomic alterations and their impact on affected signaling pathways (Section 2.3.1). ClinOmicsTrail^bc^ also reviews the standard-of-care breast cancer drugs with respect to a variety of relevant factors, such as the status of the molecular drug targets, drug-processing enzymes, transporters and involved pathways (Section 2.3.2). Besides, putative candidates for drug repositioning are indicated and can be further investigated. Additionally, the potential suitability of immunotherapies is determined with respect to neoepitope vaccines and checkpoint inhibitors (Section 2.3.3). Finally, ClinOmicsTrail^bc^ performs a first assessment of the patient’s eligibility to participate in clinical trials (Section 2.3.4).

#### 2.3.1 Overview of specific tumor characteristics

**Fig. 4.**
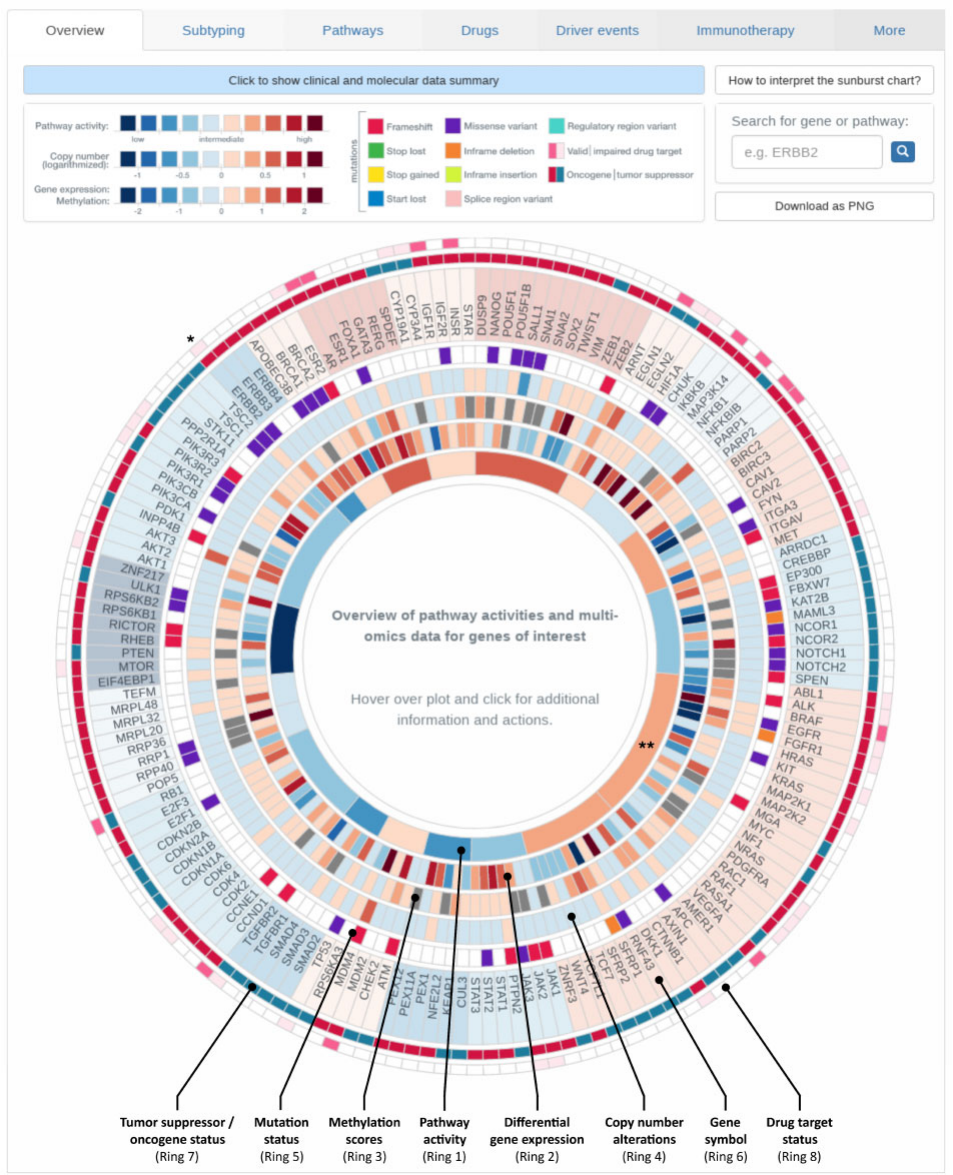
Overview of tumor characteristics. Breast cancer-relevant driver genes and pathways are displayed in a circular manner. Genes are grouped according to the pathways they are most characteristic for. The plot is organized in rings, where the innermost ring displays pathway activities, the second ‘inner’ ring corresponds to gene expression. Depending on the data provided by the user, information on copy number alterations, methylation and mutations is shown in the third, fourth and fifth ring respectively. Gene names are displayed in the next ring. The second most outer ring indicates whether the gene acts as an oncogene or tumor suppressor gene (TSG) for activating the corresponding pathway. The outermost ring contains indicators on whether or not the gene is a known drug target. Visualization for sample TCGA-BH-A0DT (cf. Section 3.2). * Entry on HER2/neu (ERBB2), ** MAPK signaling pathway as referred to in Section 3.2

ClinOmicsTrail^bc^ provides a comprehensive overview of a tumor’s specific characteristics in the form of a sunburst chart (Fig. 4). Relevant signaling pathways and a selection of their driver genes and other genes mirroring the pathway activity are displayed in a circular manner. The innermost ring represents cancer-relevant pathways. Each pathway (segment) is colored by its inferred activity. Clicking on a pathway of interest zooms into this pathway for a focused representation of the data. Depending on the types of provided omics data, up to seven additional rings are displayed: in the most comprehensive case the rings indicate (from inside out) sample specific measurements of (i) differential gene expression, (ii) (differential) methylation scores, (iii) copy number alterations and (iv) genomic mutations, (v) the corresponding gene’s name and indicators on whether the gene is an (vi) oncogene or a tumor suppressor as well as its (vii) druggability status. The sunburst chart is also connected to various third-party resources allowing for a detailed investigation of specific genes or pathways. For example, clicking on a gene’s name opens details from NCBI Gene, whereas selecting the druggability indicator yields additional information on its targeting drug(s) as obtained from DrugBank (Law *et al.*, 2014). Additional details on the specific mutations and various scores indicating their severity (SIFT and PolyPhen) can be obtained by clicking on a mutation of interest.

This interactive overview is fully searchable, zoomable and extendable. Searching for a gene or pathway of interest will highlight the respective section in the plot. Moreover, the user can interactively add genes of interest that are not yet contained in the sunburst chart by entering the gene’s name into the search field. The respective entries will be appended to the visualization in a user-defined category.

#### 2.3.2 Assessment of targeted therapies

**Fig. 5.**
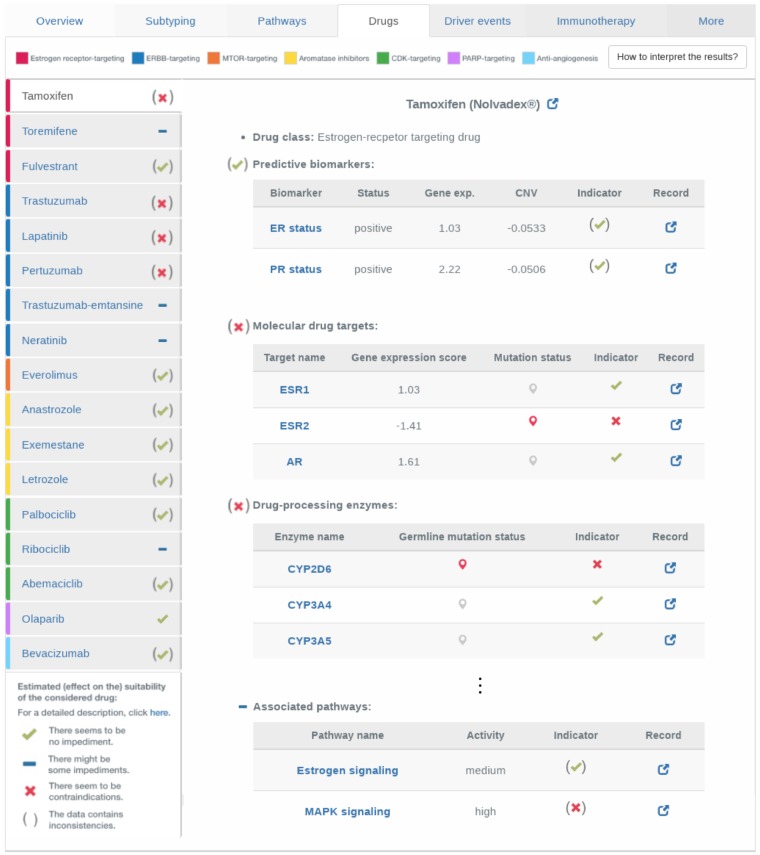
Assessment of recommended drugs. For a set of 17 standard-of-care breast cancer drugs (left panel), various factors increasing or decreasing the efficacy of a drug are assessed. Clinical, genetic and molecular characteristics are listed with an indicator sign on whether they might decrease efficacy or even cause resistance to the treatment with the drug under consideration. All genes and pathways are linked to third-party resources, where additional details can be found. Each entry also contains the link to a record or publication that describes the role of the corresponding gene with respect to the drug of interest. Here, the results for the exemplary sample TCGA-BH-A0DT are displayed (cf. Section 3.2). The pathway activity label ‘medium’ corresponds to pathway activity scores in [0.4, 0.6] and ‘high’ to pathway activity scores in (0.6, 1]

For a set of 17 FDA-approved, standard-of-care breast cancer drugs (Fig. 5), ClinOmicsTrail^bc^ assesses the genomic and transcriptomic status of respective molecular drug targets, drug-processing enzymes, resistance-promoting factors and associated pathways. Relevant factors to consider were obtained from DrugBank (Law *et al.*, 2014), the Therapeutic Target Database (Li *et al.*, 2018) and the literature. Since the respective categories reflect different mechanisms that might (de)sensitize a tumor with regard to the considered drug, different clinical, genomic and transcriptomic traits have to be considered in each case. For many breast cancer drugs, there are well-known predictive biomarkers that inform the treatment decision-making process. In ClinOmicsTrail^bc^, predictive biomarkers like ER status, PR status or HER2/neu amplification status are evaluated first and foremost on the clinical data provided by the user. However, these clinical indications are also compared to gene expression and/or copy number data to spot potential inconsistencies.

Another important set of factors for drug efficacy are the molecular drug target(s) of a compound of interest. Here, it is favorable if the drug target is highly expressed in the tumor. ClinOmicsTrail^bc^ also investigates whether the drug target contains a mutation and, if this is the case, assesses the mutation’s severity based on SIFT, PolyPhen and VeP impact scores to determine if the target might have attained a resistance mutation.

When investigating putative drug efficacy, altered pharmacokinetic mechanisms also are a major resource of variability in treatment response (Božina *et al.*, 2009). In this regard, ClinOmicsTrail^bc^ assesses drug-metabolizing enzymes as well as efflux transporters. Many drugs require an activation by drug-metabolizing enzymes like cytochromes in the liver (Zanger and Schwab, 2013). ClinOmicsTrail^bc^ uses the (germline) mutation status of the respective enzymes to determine whether or not drugs can be metabolized to their active forms. For efflux transporters, ClinOmicsTrail^bc^ especially assesses their gene expression to detect increased activity, but it also inspects somatic mutations.

As a final class of modulators of drug response, we consider whole signaling pathways. Here, pathways directly targeted by the compound under consideration should show strong activities in the tumor. This ensures that the drug actually tackles a disease-driving mechanism. A summary of the rule-based system for drug evaluation is provided in Supplementary File S5.

Besides the assessment of on-label drugs, ClinOmicsTrail^bc^ also investigates a set of 23 ‘driver targeting drugs’, i.e. drugs that require the presence or absence of pathological markers, mutations or other genomic alterations. Due to the fact that these drugs are approved for various cancer types, however not necessarily for breast cancer, they can be considered as off-label treatment options. In order to determine whether or not a patient could be stratified for the administration of the respective drugs, ClinOmicsTrail^bc^ evaluates the genomic alterations in the tumor with respect to specific point mutations, copy number alterations, transcriptomic deregulation and hormone receptor status.

#### 2.3.3 Immunotherapy assessment

**Fig. 6.**
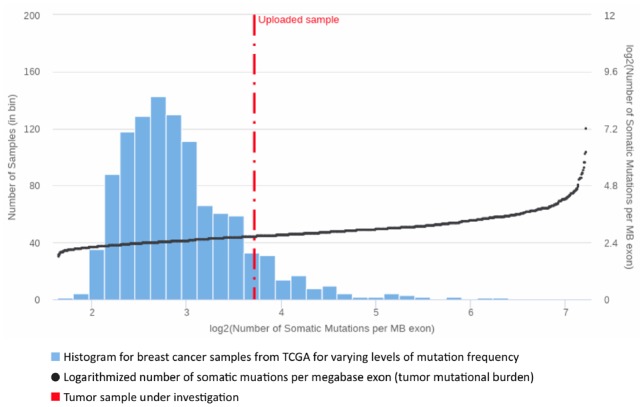
Tumor mutational burden. Visualization of the tumor mutational burden for a sample of interest (TCGA-A2-A0T2, cf. Section 3.3) in comparison to the TCGA breast cancer cohort. The bars indicate the number of TCGA samples per interval of mutation frequencies (left y-axis). The TCGA samples are sorted by increasing mutation load. The black dots depict the logarithmized number of somatic mutations per megabase exon (right y-axis)

One of the most dynamic areas of innovation in cancer treatment is immunotherapy, which aims at (re-)enabling the immune system to recognize and destroy cancerous cells (Couzin-Frankel, 2013). Many studies show that tumors with a high mutational load have the potential to respond well to immune system stimulating therapies like checkpoint inhibitors, antigen vaccination or adoptive cell therapy (Goodman *et al.*, 2017; Lauss *et al.*, 2017). For the evaluation of these treatment options, ClinOmicsTrail^bc^ determines the tumor’s mutational burden (TMB) as the total number of somatic mutations per megabase exon in the genome (Goodman *et al.*, 2017). The TMB of a considered tumor is then displayed in comparison to the TCGA breast cancer cohort (Fig. 6). As high mutation rates are oftentimes caused by deficiencies in the DNA repair machinery, ClinOmicsTrail^bc^ also assesses the status of a variety of repair genes (MD Anderson Cancer Center, 2018). Additionally, ClinOmicsTrail^bc^ provides an overview on the (epi-)genomic and transcriptomic status of a variety of genes serving as biomarkers for checkpoint inhibitors.

Besides checkpoint blockade, personalized cancer vaccines are another promising approach to cancer immunotherapy (Ott *et al.*, 2017; Sahin *et al.*, 2017). Cancer vaccines target overexpressed or altered proteins and HLA presented peptide sequences (neoepitopes) that resulted from somatic mutations uniquely characterizing the patient’s tumor. They are used to prime T cells to recognize these characterizing antigens and destroy the presenting tumor cells. As the neoepitopes are dependent on both, the patient’s tumor mutations and HLA genotype, cancer vaccines have to be individually designed. To this end, ClinOmicsTrail^bc^ offers functionalities to predict potential neoepitope vaccine targets based on the identified somatic mutations and HLA genotype of a patient using the immunoinformatic toolbox ImmunoNodes (Schubert *et al.*, 2017). It provides various classes of epitope prediction methods to compute (neo-)epitopes and to assess their affinity to the patient’s HLA genotype. Supplementary File S6 contains additional details on the 13 provided methods. The identified epitopes can then serve as basis for the synthesis of a personalized cancer vaccine, which could also be combined with checkpoint inhibitors to boost the vaccine’s effectiveness (Fu *et al.*, 2014).

#### 2.3.4 Clinical trial matching

In cases where standard-of-care treatment solutions are not applicable, it might be of interest to examine potential clinical trials the patient is eligible to enroll in. To this end, ClinOmicsTrail^bc^ links to phase II, III and IV clinical trials registered in ClinicalTrials.gov and the European Union Clinical Trials Register, which are recruiting in many countries. Additionally, ClinOmicsTrail^bc^ makes a first assessment of the eligibility for various classes of clinical trials listed on BreastCancerTrials.org. This stratification considers tumor characteristics like the BRCA1/2 mutation status and the tumor grade, as well as different treatment types including hormone therapy, PARP inhibitors, targeted therapy and immunotherapy.

#### 2.3.5 Investigation of deregulated drug targets and signaling processes

For an even deeper investigation of deregulated drug targets and altered signaling processes in the tumor, ClinOmicsTrail^bc^ is natively integrated with its sister projects DrugTargetInspector (Schneider *et al.*, 2016) and GeneTrail2 (Stöckel *et al.*, 2016). DrugTargetInspector is a web server for the interactive investigation of drug targets and dysregulated signaling pathways. GeneTrail2 is a web-interface providing access to different tools for the statistical analysis of molecular signatures with a focus on pathway enrichment analyses. It offers multiple statistical tests and a comprehensive collection of biological pathways. Once omics datasets are uploaded to ClinOmicsTrail^bc^, the functionality of both tools is readily available.

## 3 Results

In order to demonstrate ClinOmicsTrail^bc^‘s capabilities to support the treatment decision-making process, we present three case studies that showcase how ClinOmicsTrail^bc^ may guide the treatment selection process by identifying pathway activity patterns driving the tumor under investigation (Section 3.1), assessing a set of drugs approved for breast cancer treatment by an in-depth investigation of modulators of treatment success (Section 3.2), and evaluating predictive biomarkers for immunotherapy as a treatment option (Section 3.3). Supplementary Figures and links to the interactive results pages for the respective case studies are provided in Supplementary File S7.

### 3.1 Pathway activity patterns guiding treatment selection

As current standard of care, breast cancer subtypes are typically determined based on a few pathological markers or small-scale gene expression profiles like the PAM50 classifier (Parker *et al.*, 2009). Here, we demonstrate that the consideration of a broader spectrum of gene expression in the form of pathway activity patterns allows to draw a more differentiated picture of the tumor’s intrinsic subtype, thereby guiding the treatment decision-making process.

For illustratory purposes, we considered the tumor sample of a 68-year-old woman (TCGA-AN-A0XN) with a stage III breast cancer (ER negative, PR positive, HER2 negative) that was classified as the luminal A subtype by PAM50. ClinOmicsTrail^bc^ provides functionality for the clustering of the sample under investigation into a ‘neighborhood’ of TCGA breast tumor samples with similar gene expression signatures (‘Subtyping’ tab), allowing for an assessment of the tumor’s subtype based on its complete gene expression profile. For our sample of interest, we can observe that, although it was predicted to be of the luminal subtype, it also has similarities to the HER2-enriched and basal-like subtypes (cf. Fig. 3).

For a more granular investigation of the tumor’s characteristics, ClinOmicsTrail^bc^ computes the pathway activities of a set of 20 core cancer-relevant pathways that can exhibit subtype-specific patterns. The pathway activities depicted in the ‘Pathways’ view show that in our sample of interest—in contrast to the majority of breast tumors—the PI3K-Akt signaling pathway seems to be inactive. However, several pathways related to stem cell characteristics (e.g. Focal adhesion and HIF-1 signaling) seem to be strongly upregulated (cf. Fig. 2).

The activation of these pathways has already been shown to be characteristic for basal-like tumors (Golubovskaya *et al.*, 2014; Thiagarajan *et al.*, 2018). ClinOmicsTrail^bc^ shows that the pathway activity pattern of the considered sample is very similar to the one of the triple-negative, basal-like TCGA sample TCGA-BH-A18G, see Figure 2. Furthermore, the ten cell lines most similar to our tumor sample of interest are all triple-negative (cf. Supplementary File S7).

The investigation of these pathway activity patterns might also reveal targets for possible therapeutic intervention. In the considered sample, the MAPK signaling pathway is strongly activated, most likely due to the upregulation of key pathway components like BRAF, KRAS and NRAS. Therapeutic intervention in this pathway might hence be an option. This finding is supported by the fact that the two cell lines most similar to the sample under investigation regarding pathway activity patterns (SUM149PT and 185B5) are known to be sensitive against an ERK inhibitor. Another potential option suggested by ClinOmicsTrail^bc^ might be a treatment with bevacizumab as its molecular target, the vascular epithelial growth factor (VEGFA) (Ranieri *et al.*, 2006), is strongly upregulated (z-score = 3.63).

### 3.2 Assessment of standard-of-care breast cancer drugs

The selective estrogen receptor modifier tamoxifen is one of the oldest and most commonly prescribed breast cancer drugs. However, more than 30% of patients with adjuvant tamoxifen treatment relapse or die (Schroth *et al.*, 2007). This is likely due to de-novo or acquired tamoxifen resistance that can be mediated by a variety of genetic and molecular factors. In order to exemplify ClinOmicsTrail^bc^‘s thorough assessment of these factors, we consider the tumor sample of a 41-year-old woman with a stage II, hormone receptor-positive, HER2-negative breast tumor (TCGA-BH-A0DT). As the considered sample is hormone receptor positive and HER2 negative, tamoxifen might be considered as treatment of choice. In order to obtain a more comprehensive picture, ClinOmicsTrail^bc^ provides a ‘Drugs’ view that contains information on the status of several biomarkers potentially relevant to estimate treatment success like molecular targets, drug-processing enzymes and transporters (cf. Fig. 5).

Our sample under investigation exhibits several alterations that are likely to cause resistance against tamoxifen. In order for tamoxifen to be metabolized into its active form endoxifen, the cytochrome P450 family member CYP2D6 is required (Goetz, 2009). For our given sample, ClinOmicsTrail^bc^ identified a frameshift variant in CYP2D6 that most likely generates a poor metabolizer phenotype and hence contributes to a potential resistance against tamoxifen.

Besides the androgen receptor (AR), tamoxifen also targets the estrogen receptors 1 and 2 (ESR1/ERα, ESR2/ERβ), a family of transcription factors that are activated by estrogens, mediating the activation of a variety of growth-promoting processes (Ascenzi *et al.*, 2006). In our sample under investigation, ESR2 contains a frameshift variant that is likely to affect the protein’s structure severely and hence might drastically reduce tamoxifen’s affinity to its target ESR2.

Other relevant factors are co-regulators of ER-mediated transcription. One such regulatory element is the cytosine deaminase APOBEC3B, which typically deaminates cytosine to uracil in ER enhancer regions, thereby activating base excision repair pathways, which in turn promote chromatin remodeling that enables the expression of ER target genes (Periyasamy *et al.*, 2015). Higher levels of APOBEC3B expression have been associated with poor clinical outcome of tamoxifen treatment in ER-positive breast cancer (Law *et al.*, 2016). In our sample of interest, we can observe an increased level of APOBEC3B expression, serving as further evidence in disfavor of tamoxifen.

Finally, the considered sample shows increased levels of MAPK signaling pathway activity and an upregulation of HER2/neu (ERBB2) (cf. Fig. 4), which might contribute to resistance against endocrine therapy via the ligand-independent activation of ER through ERK (Ring and Dowsett, 2004).

To summarize, although the clinical data for the considered sample might point towards a treatment with tamoxifen, a broad investigation of molecular determinants of treatment success could highlight several factors that might render tamoxifen ineffective in the considered case.

Besides selective estrogen receptor modulators like tamoxifen, ClinOmicsTrail^bc^ also provides an in-depth assessment of the most relevant targeted drug classes for breast cancer treatment (cf. Fig. 5). Although we could observe an upregulation of ERBB2 expression in our sample under investigation, trastuzumab and other ERBB-targeting drugs might be impeded by a missense variant in ERBB2. This mutation could reduce the efficacy of this class of drugs, despite the fact that it has been classified to only have a moderate impact on the protein’s structure. As indicated by ClinOmicsTrail^bc^, a putative treatment option for our investigated sample might be the use of aromatase inhibitors like anastrozole, exemestane or letrozole. As aromatase inhibitors are typically prescribed to postmenopausal women (Fabian, 2007), a successful treatment will require additional ovary suppression, which has been shown to significantly improve response rates in premenopausal women in the SOFT trial (Francis *et al.*, 2015). Also, since our sample of investigation contains BRCA1/2 germline mutations and the poly-ADP-ribosyl-transferase PARP1 is strongly upregulated (z-score = 4.99), PARP inhibitor treatment is suggested as potential option by ClinOmicsTrail^bc^.

### 3.3 Impaired DNA repair informs personalized immunotherapy

As an example showcasing ClinOmicsTrail^bc^’s capabilities for decision support in immunotherapy, we considered the tumor sample of a 66-year-old woman with a stage IV, triple-negative, metastatic breast cancer (TCGA-A2-A0T2). The sample exhibits a rather high tumor mutational burden of 13.18 somatic mutations per megabase exon as shown in the ‘Mutational burden’ tab (cf. Fig. 6). ClinOmicsTrail^bc^’s analysis of potential driver genes in the ‘Driver events’ view yields additional insights into which factors might be causing this high mutation load. Here, the analysis revealed severe and probably damaging mutations in TP53, RB1 and ATM. The transcription factor TP53 is an essential tumor suppressor that is commonly compromised in human cancers. The encoded protein is involved in a variety of cellular processes, including cell cycle arrest, senescence, apoptosis and DNA repair (Levine *et al.*, 1991). Similarly, RB1 acts as tumor suppressor by negatively regulating the cell cycle. It is also involved in stabilizing heterochromatin, thereby maintaining the overall chromatin structure (Gonzalo *et al.*, 2005). Hence, alterations in RB1 can cause genomic instability, thereby fostering an accumulation of mutations and providing an evolutionary advantage to the affected cancer cells (Palmieri *et al.*, 2017). Finally, ATM is an important cell cycle checkpoint kinase that regulates various tumor suppressors like TP53 or BRCA1, acting as key regulators governing genome stability and response to DNA damage (Matsuoka *et al.*, 2007).

Besides known driver mutations, ClinOmicsTrail^bc^ also checks the mutation status of a variety of genes involved in the DNA repair machinery and provides an overview of potentially impaired genes in the ‘Repair genes’ tab of the ‘Immunotherapy’ view. For our sample under investigation, these results revealed a variety of mostly damaging mutations in various components of DNA repair (Supplementary File S8).

Taken together, the mutational burden in combination with the likely impairment of repair mechanisms might render checkpoint blockade (potentially in combination with DNA-damaging agents or neoepitope vaccination) an effective treatment strategy in this case (Wein *et al.*, 2018). To further assist the selection of a suitable checkpoint inhibitor, ClinOmicsTrail^bc^ provides an overview of the genomic and transcriptomic features of various druggable checkpoint genes. With respect to personalized cancer vaccines, ClinOmicsTrail^bc^ identifies tumor-specific neoepitopes that can serve as basis for vaccine development (see Supplementary File S9).

## 4 Discussion and conclusions

We presented ClinOmicsTrail^bc^, a powerful visual analytics tool for breast cancer treatment stratification. Our tool supports precision medicine (i) by assessing and prioritizing standard-of-care breast cancer drugs, (ii) by suggesting drugs for off-label use, (iii) by evaluating the potential of different types of immunotherapy including checkpoint inhibitors and personalized cancer vaccines, and (iv) by assessing a patient’s eligibility to enroll in clinical trials. To this end, ClinOmicsTrail^bc^ performs a multitude of analyses on a tumor’s clinical markers and (epi-)genomic and transcriptomic alterations to systematically characterize the tumor with respect to its main driver mutations, its mutational burden, the activity patterns of cancer-relevant pathways and the status of drug-specific predictive biomarkers, molecular drug targets and involved ADME genes.

In order to optimally support clinicians, conciseness and interpretability of the results are essential. For that purpose, ClinOmicsTrail^bc^ summarizes key tumor characteristics and additional information on drug-specific biomarkers and modulators of treatment response in a few comprehensive, yet easily interpretable visualizations providing clinicians with the most relevant information at the point of care. Furthermore, we provide an extensive documentation of the web service, ranging from stand-alone tutorials over additional help and information along the data upload and analysis steps to interactive explanations of the provided results.

ClinOmicsTrail^bc^ is an ongoing project, which we will further extend and continue developing. For upcoming versions, we plan to incorporate further databases [e.g. CIViC (Griffith *et al.*, 2017), OncoKB (Chakravarty *et al.*, 2017), Cancer Genome Interpreter (Tamborero *et al.*, 2018)] to continuously improve the annotation of aberrations in a tumor with pharmacogenomic effects on drug sensitivity. Furthermore, we also plan to provide additional reference datasets besides TCGA, e.g. multi-omics reference data from the International Cancer Genome Consortium (Zhang *et al.*, 2011). With respect to reference data, it would also be desirable to set up a collection of gene expression measurements of healthy control samples (obtained via different types of experimental platforms) to serve as references in cases where no matched normal sample for a tumor is available.

Albeit ClinOmicsTrail^bc^ is optimized for the analysis of breast cancer datasets, the underlying analysis methods and visualization techniques offered by our web service can also be used for the genetic and molecular characterization of other tumor types by mainly exchanging the tumor-specific underlying databases. We plan to provide adapted versions for other tumor types in the near future.

The breadth and depth of analyzes and visualizations offered by ClinOmicsTrail^bc^ make it – although being in a proof-of-principle stage - an indispensable addition to the existing clinical decision support machinery. The three presented case studies convey a first impression on the capabilities of the tool, however can only partly illustrate its full potential. From a clinical perspective, ClinOmicsTrail^bc^ is a comprehensive tool suite that will be further validated regarding its benefits in the preparation and conduction of molecular tumor board meetings.

In summary, ClinOmicsTrail^bc^ is a powerful integrated visual analytics tool for breast cancer research in general and for therapy stratification in particular, assisting oncologists to find the best possible treatment options in a deeply personalized way.

## Funding

This work was supported by Deutsche Forschungsgemeinschaft [LE952/3-2]. C.M. and O.K. acknowledge funding from the European Union Horizon 2020 Framework (APERIM, contract no. 633592).

*Conflict of Interest:* A.H. has received honoraria from Novartis, Pfizer, MSD, AstraZeneca, Roche, Eisai, GenomicHealth and Lilly. M.W. has received honoraria from Celgene, Amgen and AstraZeneca. The other authors declare no potential conflicts of interest.

## Supplementary Material

btz302_Supplementary_Data
