# Supplementary material for: ClinOmicsTrailbc: a visual analytics tool for breast cancer treatment stratification
Source: Bioinformatics. 2019 Apr 30;35(24):5171–81. doi: 10.1093/bioinformatics/btz302 (PMC6954665; doi:10.1093/bioinformatics/btz302)
Supplement: btz302_Supplementary_Data [file btz302_supplementary_data.zip › btz302-Suppl_data/Supplementary_Data_S4.pdf]

Pathway activity computation

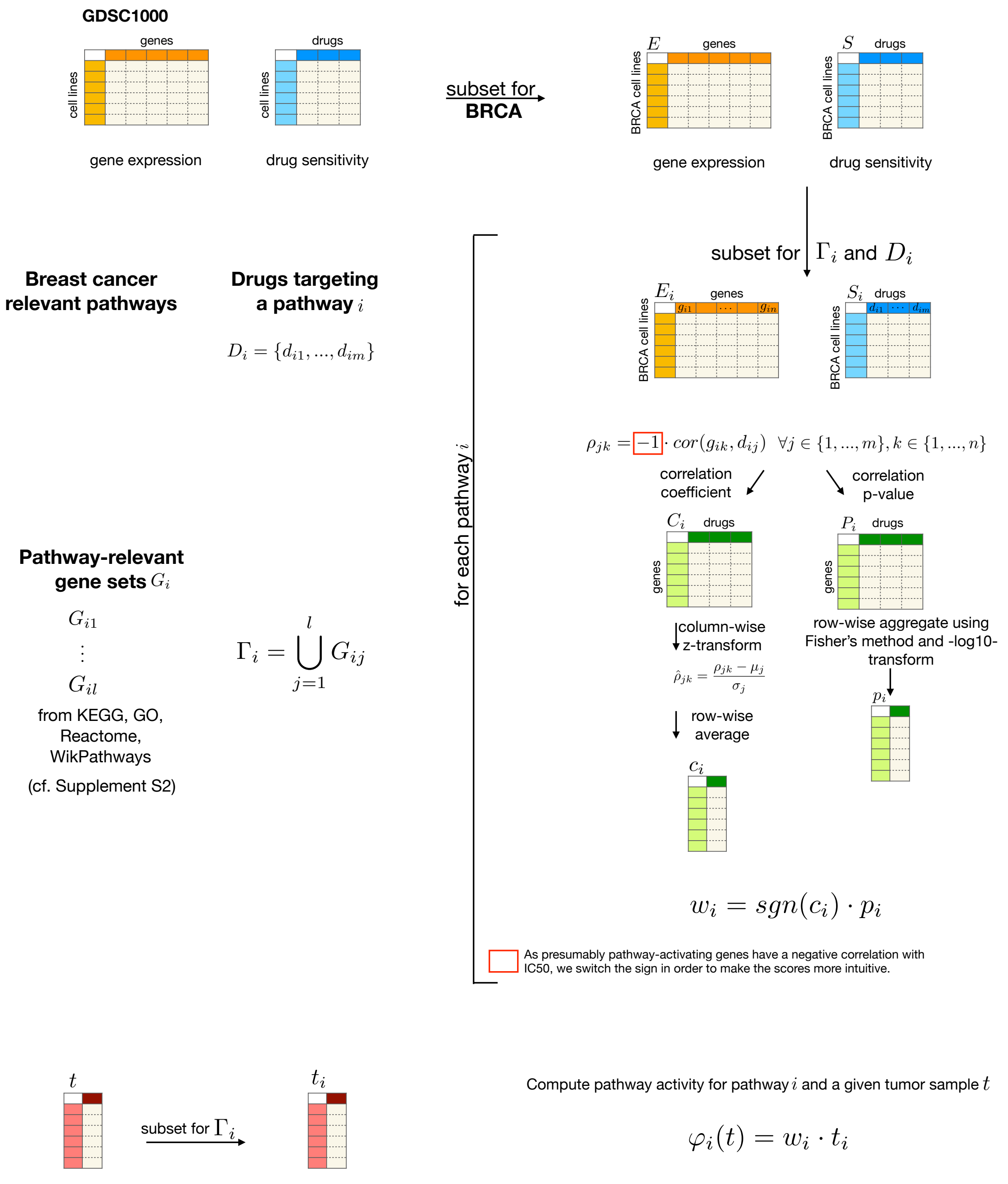

Figure 1. Overview of pathway activity computation.

Assessment of pathway activities

Tumors are driven by the aberrant, increased or decreased, activity of key signaling pathways that e.g. promote tumor growth or hinder apoptosis[1]. In order to get an overview of altered processes in a breast tumor under investigation, we consider pathway activities of a set of 20 breast cancer-relevant pathways[2-4]. These patterns can in turn be used to assess characteristics of tumor subtypes and to inform a treatment decision, as for example tumor with high activities in PIK-AKT-mTOR signaling would profit from treatment with e.g. the mTOR inhibitor everolimus[5].

As a proxy for the actual pathway activities, we compute the deregulation of metagenes for each pathway i. Here, a metagene is based on the deregulation of the set of pathway-constituting genes Γ_i, weighted by their relevance $\boldsymbol{w_i}$ for the respective pathway's activity. In order to obtain a comprehensive set of genes involved in the activity of a given pathway, we merge the gene sets of relevant (sub-)pathways and biological categories provided by KEGG[6], GO[7], Reactome[8], and WikiPathways[9], see Supplement S2 for details. This merging step helps to counteract the incompleteness and variability of descriptions of pathways and functional categories in current databases.

We hypothesize that targeted drugs are especially effective in cases where their target pathway is highly active and alternative cancer-driving pathways are not[10]. We take advantage of the assumed relationship between a pathway's activity and a corresponding drug's efficacy to compute the weights w_i. To this end, we consider all 49 breast cancer cell lines from the Genomics of Drug Sensitivity in Cancer (GDSC1000) database[11] and their sensitivities for a large panel of drugs targeting various pathways. The authors provided drug sensitivity scores as IC50 values, i.e. the 10*log10-transformed concentration of an inhibitor that decreases the biotransformation rate of its target's substrates by 50%[12]. For a given pathway of interest i, we select the set D_i of drugs from GDSC that target this pathway. For each of those drugs $d_{ij} \in D_i$, we compute Pearson's correlation[13] between the drug's IC50 values and the gene expression measurements across cell lines. For each of those correlation coefficients ρ_{jk}, we also compute a p-value assessing the significance of its deviation from zero. This results in two matrices of dimensions $n_i \times m_i$ each, where n_i corresponds to the size of the gene set Γ_i and m_i to the size of the drug set D_i. The matrices are then transformed as follows: the correlation coefficients are z-transformed per drug and then averaged cross drugs yielding a list of correlation-based scores per pathway $\boldsymbol{c_i}$. The p-values are aggregated across drugs using Fisher's method[14]. The aggregated p-values are then -log10-transformed to obtain scores per pathway and gene $\boldsymbol{p_i}$. The larger the score p_{ik} for a gene k, the more relevant it is as an indicator for the pathway's activity. As the scores in $\boldsymbol{p_i}$ are all positive, we recover the direction of the gene's effect, i.e. whether it acts as an activator or repressor of the pathway, from the sign of the corresponding correlation-based score c_{ik}. The final weights $\boldsymbol{w_i}$ are then computed as $\boldsymbol{w_i} = sgn(\boldsymbol{c_i}) \cdot \boldsymbol{p_i}$. The pathway activity $\varphi_i(\boldsymbol{t})$ for a tumor sample $\boldsymbol{t}$ and a pathway i is then computed as $\varphi_i(\boldsymbol{t}) = \boldsymbol{w_i} \cdot \boldsymbol{t_i}$, where $\boldsymbol{t_i}$ is the subset for the genes in the gene set Γ_i. Pathway activities per pathway finally are embedded into a range between 0 and 1.

In order to assess the significance of the computed pathway activities, empirical p-values are derived using permutation testing. Based on a user-defined number of permutations, the sample's scores of differential gene expression are randomly permuted and the corresponding pathway activities are re-computed. One-sided p-values are then computed relative to the mean of the empirical background distribution, where a right-sided p-value is computed if the sample's pathway activity is larger than the mean of the background distribution and a left-sided p-value if it is smaller. In a last step, the derived p-values are adjusted for multiple hypothesis testing using the Benjamini-Hochberg method[15].

References

1. Giancotti, F. G. Deregulation of Cell Signaling in Cancer. *FEBS letters* **588,** 2558–2570 (2014).
2. Velloso, F. J. *et al.* The crossroads of breast cancer progression: insights into the modulation of major signaling pathways. *OncoTargets and therapy* **10,** 5491–5524 (2017).
3. Luo, M. & Guan, J.-L. Focal adhesion kinase: A prominent determinant in breast cancer initiation, progression and metastasis. *Cancer Letters* **289,** 127–139 (2010).
4. Africander, D. & Storbeck, K.-H. Steroid metabolism in breast cancer: Where are we and what are we missing? *Molecular and Cellular Endocrinology* **466,** 86–97 (2018).
5. Porta, C., Paglino, C. & Mosca, A. Targeting PI3K/Akt/mTOR Signaling in Cancer. *Frontiers in Oncology* **4,** 2905 (2014).
6. Kanehisa, M., Sato, Y., Kawashima, M., Furumichi, M. & Tanabe, M. KEGG as a reference resource for gene and protein annotation. *Nucleic Acids Research* **44,** D457–D462 (2016).
7. Ashburner, M. *et al.* Gene ontology: tool for the unification of biology. The Gene Ontology Consortium. *Nature Genetics* **25,** 25–29 (2000).
8. Fabregat, A. *et al.* The Reactome Pathway Knowledgebase. *Nucleic Acids Research* **46,** D649–D655 (2018).
9. Kutmon, M. *et al.* WikiPathways: capturing the full diversity of pathway knowledge. *Nucleic Acids Research* **44,** D488–D494 (2016).
10. Amadoz, A., Sebastian-Leon, P., Vidal, E., Salavert, F. & Dopazo, J. Using activation status of signaling pathways as mechanism-based biomarkers to predict drug sensitivity. *Scientific Reports* **5,** 18494 (2015).
11. Iorio, F. *et al.* A Landscape of Pharmacogenomic Interactions in Cancer. *Cell* **166,** 740–754 (2016).
12. Bachmann, K. in *Pharmacology* 303–325 (Elsevier, 2009). doi:10.1016/B978-0-12-369521-5.00012-9
13. Pearson, K. in *Proceedings of the Royal Society of London* (1895).
14. Fisher, R. A. *Statistical Methods for Research Workers*. (Oliver and Boyd, Edinburgh, 1958).
15. Benjamini, Y. & Hochberg, Y. Controlling the False Discovery Rate: A Practical and Powerful Approach to Multiple Testing. *Journal of the Royal Statistical Society* **57,** 289–300 (1995).
