# Supplementary material for: ClinOmicsTrailbc: a visual analytics tool for breast cancer treatment stratification
Source: Bioinformatics. 2019 Apr 30;35(24):5171–81. doi: 10.1093/bioinformatics/btz302 (PMC6954665; doi:10.1093/bioinformatics/btz302)
Supplement: btz302_Supplementary_Data [file btz302_supplementary_data.zip › btz302-Suppl_data/Supplementary_Data_S5.pdf]

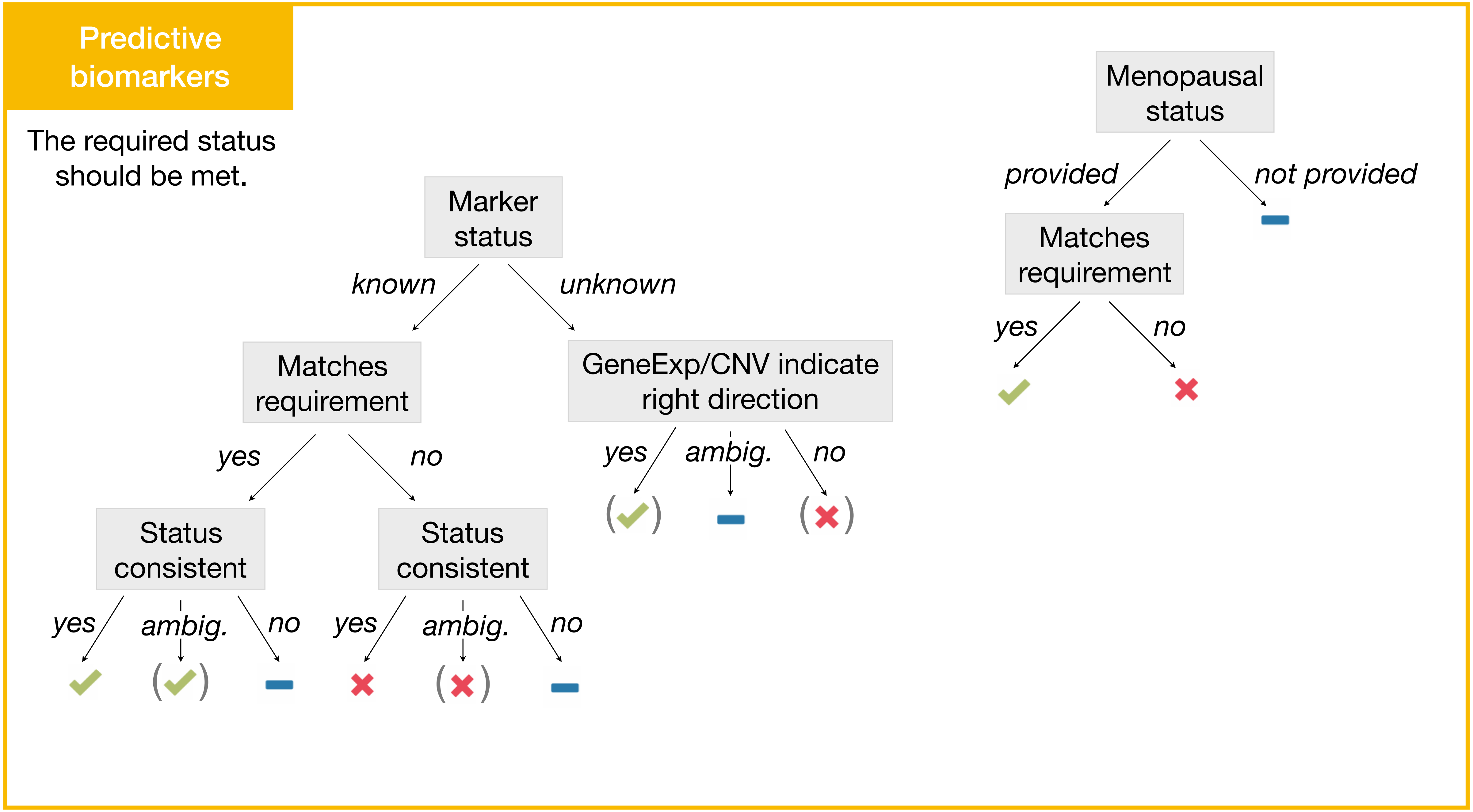


✓ There seems to be no impediment.

(✓) There seems to be no impediment, but there are inconsistencies in the data.

– There might be some impediments.

(✗) There seem to be contra-indications, but there are inconsistencies in the data.

✗ There seem to be contraindications.

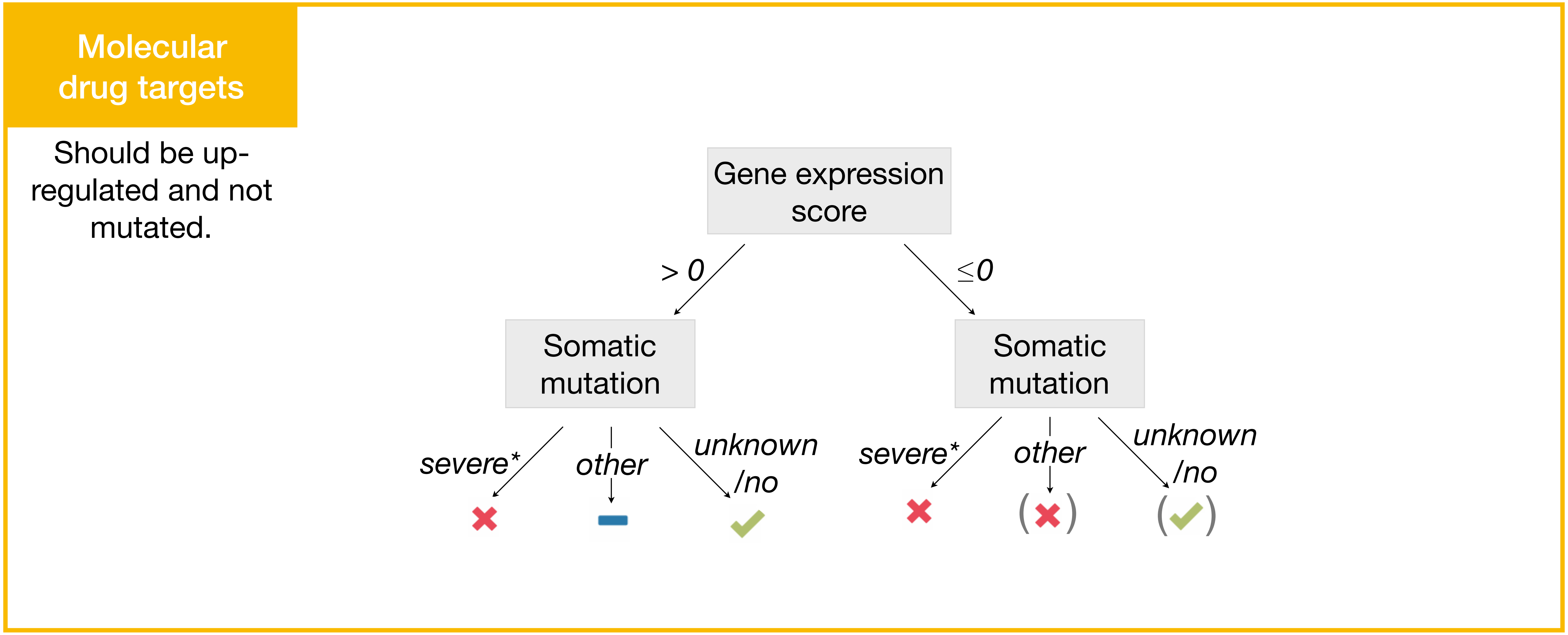


*Severe mutations={frameshift, stop lost, stop gained, start lost}

- There seems to be no impediment.
- There seems to be no impediment, but there are inconsistencies in the data.
- There might be some impediments.
- There seem to be contra-indications, but there are inconsistencies in the data.
- There seem to be contraindications.

## Drug-processing enzymes

They should not be germline mutated.

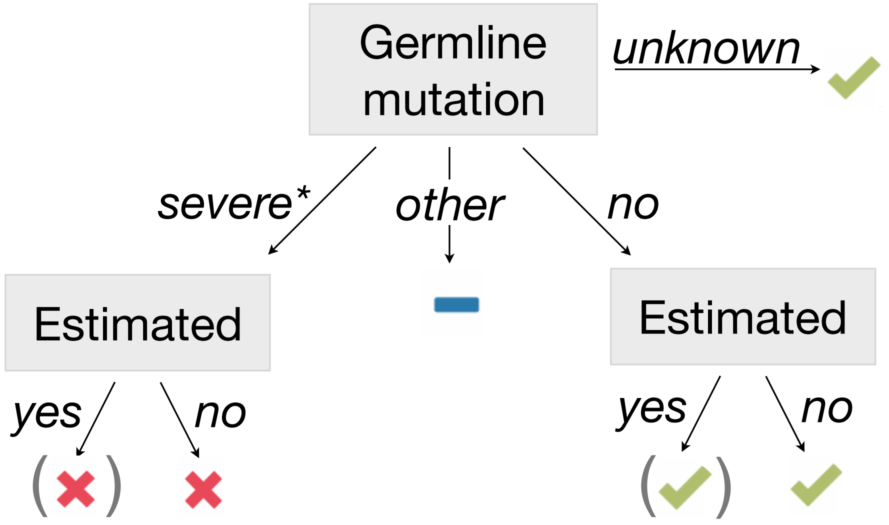


*Severe mutations={frameshift, stop lost, stop gained, start lost}

- There seems to be no impediment.
- There seems to be no impediment, but there are inconsistencies in the data.
- There might be some impediments.
- There seem to be contra-indications, but there are inconsistencies in the data.
- There seem to be contraindications.

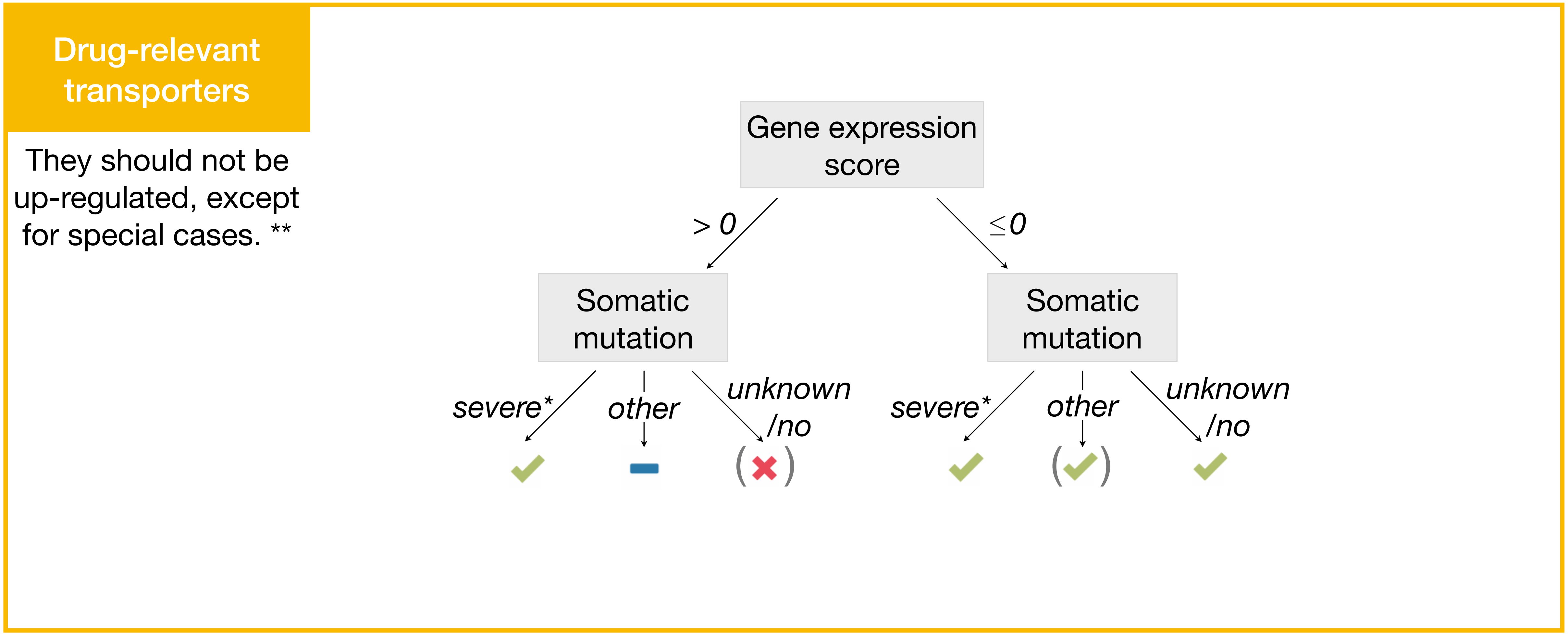


*Severe mutations={frameshift, stop lost, stop gained, start lost}

**Special cases: tamoxifen, lapatinib and abemaciclib that inhibit certain transporters.

There seems to be no impediment.

There seems to be no impediment, but there are inconsistencies in the data.

There might be some impediments.

There seem to be contra-indications, but there are inconsistencies in the data.

There seem to be contraindications.

## Associated pathways

The required status should be met.

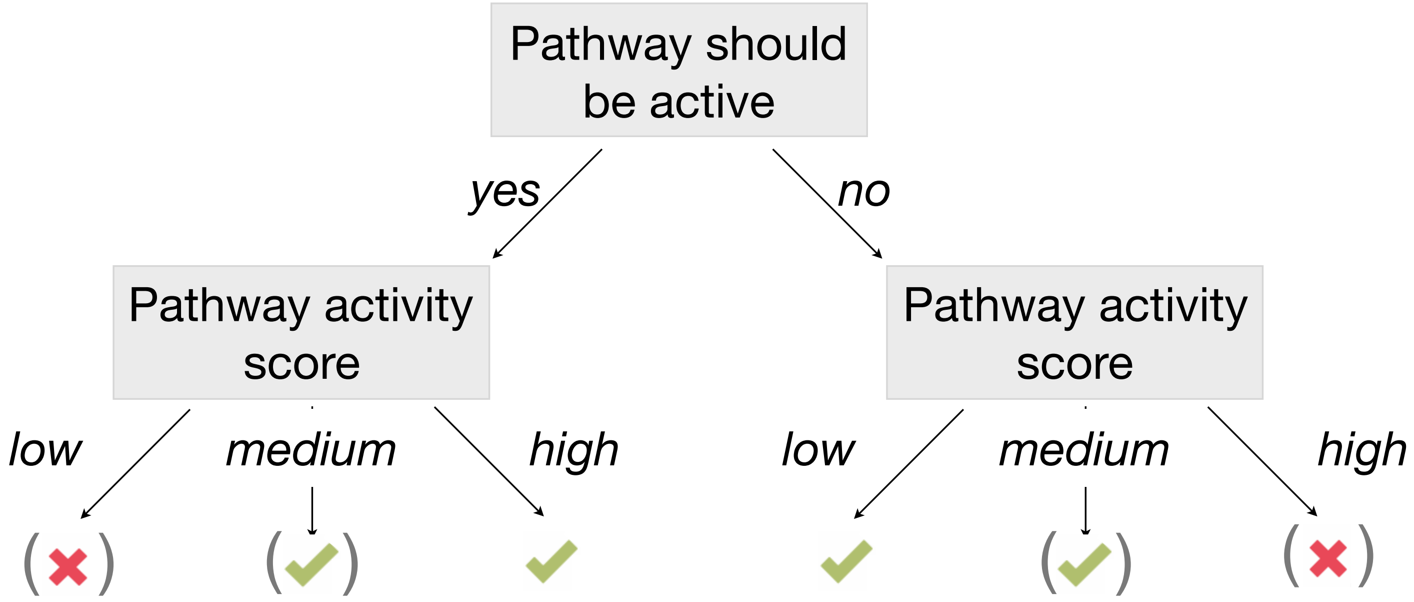


Pathway activity score in [0,0.4) => ‚low‘
Pathway activity score in [0.4,0.6] => ‚medium‘
Pathway activity score in (0.6,1] => ‚high‘

- ✔ There seems to be no impediment.
- (✔) There seems to be no impediment, but there are inconsistencies in the data.
- ▬ There might be some impediments.
- (✖) There seem to be contra-indications, but there are inconsistencies in the data.
- ✖ There seem to be contraindications.
