# Supplementary material for: ClinOmicsTrailbc: a visual analytics tool for breast cancer treatment stratification
Source: Bioinformatics. 2019 Apr 30;35(24):5171–81. doi: 10.1093/bioinformatics/btz302 (PMC6954665; doi:10.1093/bioinformatics/btz302)
Supplement: btz302_Supplementary_Data [file btz302_supplementary_data.zip › btz302-Suppl_data/Supplementary_Data_S6.pdf]

Neoepitope Prediction Methods

Besides checkpoint blockade, personalized cancer vaccines are another promising approach to cancer immunotherapy[1,2]. Cancer vaccines target overexpressed or altered proteins and HLA presented peptides sequences (neoepitopes) that resulted from somatic mutations uniquely characterizing the patient's tumor. They are used to prime T cells to recognize these characterizing antigens and destroy the presenting tumor cells. As the neoepitopes are dependent on, both, the patient's tumor mutations and HLA genotype, cancer vaccines have to be individually designed. Thus, ClinOmicsTrail[bc] offers functionalities to predict potential neoepitope vaccine targets based on the identified somatic mutations and HLA genotype of a patient using the immunoinformatic toolbox ImmunoNodes[3]. ImmunoNodes provides various classes of epitope prediction methods to compute (neo-)epitopes and to assess their affinity to the patient's set of HLA alleles. Details on the 13 methods for neoepitope prediction provided by ClinOmicsTrail[bc] are listed in Table 1.

Method	Version	Class	Reference
ARB	1.0	MHC-I binding	[4]
BIMAS	1.0	MHC-I binding	[5]
Comblib 2008	1.0	MHC-I binding	[6]
NetMHC	4.0	MHC-I binding	[7]
NetMHCII	2.2	MHC-II binding	[8]
NetMHCIIpan	3.1	MHC-II binding	[9]
NetMHCpan	3.0	MHC-I binding	[10]
PickPocket	1.1	MHC-I binding	[11]
SMM	1.0	MHC-I binding	[12]
SMMPMBEC	1.0	MHC-I binding	[13]
SVMHC	1.0	MHC-I binding	[14]
SYFPEITHI	1.0	T-cell epitope	[15]
UniTope	1.0	T-cell epitope	[16]

Table 1: Neoepitope prediction methods. This table contains the 13 neoepitope prediction methods from ImmunoNodes employed in ClinOmicsTrail[bc]. The first column contains the tools names in alphabetical order, the second column the respective version number, the third column the class of the prediction and the last column the references to the corresponding publications.

References

1. Ott, P. A. *et al.* An immunogenic personal neoantigen vaccine for patients with melanoma. *Nature* **547,** 217–221 (2017).
2. Sahin, U. *et al.* Personalized RNA mutanome vaccines mobilize poly-specific therapeutic immunity against cancer. *Nature* **547,** 222–226 (2017).
3. Schubert, B., la Garza, de, L., Mohr, C., Walzer, M. & Kohlbacher, O. ImmunoNodes – graphical development of complex immunoinformatics workflows. *BMC Bioinformatics* **18,** 242 (2017).
4. Bui, H.-H. *et al.* Automated generation and evaluation of specific MHC binding predictive tools: ARB matrix applications. *Immunogenetics* **57,** 304–314 (2005).
5. Parker, K. C., Bednarek, M. A. & Coligan, J. E. Scheme for ranking potential HLA-A2 binding peptides based on independent binding of individual peptide side-chains. *Journal of Immunology (Baltimore, Md.: 1950)* **152,** 163–175 (1994).

6. Sidney, J. *et al.* Quantitative peptide binding motifs for 19 human and mouse MHC class I molecules derived using positional scanning combinatorial peptide libraries. *Immunome Research* **4,** 2 (2008).
7. Andreatta, M. & Nielsen, M. Gapped sequence alignment using artificial neural networks: application to the MHC class I system. *Bioinformatics (Oxford, England)* **32,** 511–517 (2016).
8. Nielsen, M., Lundegaard, C. & Lund, O. Prediction of MHC class II binding affinity using SMM-align, a novel stabilization matrix alignment method. *BMC Bioinformatics* **8,** 238 (2007).
9. Karosiene, E. *et al.* NetMHCIIpan-3.0, a common pan-specific MHC class II prediction method including all three human MHC class II isotypes, HLA-DR, HLA-DP and HLA-DQ. *Immunogenetics* **65,** 711–724 (2013).
10. Nielsen, M. & Andreatta, M. NetMHCpan-3.0; improved prediction of binding to MHC class I molecules integrating information from multiple receptor and peptide length datasets. *Genome Medicine* **8,** 33 (2016).
11. Zhang, H., Lund, O. & Nielsen, M. The PickPocket method for predicting binding specificities for receptors based on receptor pocket similarities: application to MHC-peptide binding. *Bioinformatics (Oxford, England)* **25,** 1293–1299 (2009).
12. Peters, B. & Sette, A. Generating quantitative models describing the sequence specificity of biological processes with the stabilized matrix method. *BMC Bioinformatics* **6,** 132 (2005).
13. Kim, Y., Sidney, J., Pinilla, C., Sette, A. & Peters, B. Derivation of an amino acid similarity matrix for peptide:MHC binding and its application as a Bayesian prior. *BMC Bioinformatics* **10,** 394 (2009).
14. Dönnes, P. & Elofsson, A. Prediction of MHC class I binding peptides, using SVMHC. *BMC Bioinformatics* **3,** 25 (2002).
15. Rammensee, H. G., Bachmann, J., Emmerich, N. P. N., Bachor, O. A. & Stevanović, S. SYFPEITHI: database for MHC ligands and peptide motifs. *Immunogenetics* **50,** 213–219 (1999).
16. Toussaint, N. C., Feldhahn, M., Ziehm, M., Stevanović, S. & Kohlbacher, O. *T-cell epitope prediction based on self-tolerance*. *the 2nd ACM Conference* 584–588 (ACM, 2011). doi:10.1145/2147805.2147905
