# Supplementary material for: ClinOmicsTrailbc: a visual analytics tool for breast cancer treatment stratification
Source: Bioinformatics. 2019 Apr 30;35(24):5171–81. doi: 10.1093/bioinformatics/btz302 (PMC6954665; doi:10.1093/bioinformatics/btz302)
Supplement: btz302_Supplementary_Data [file btz302_supplementary_data.zip › btz302-Suppl_data/Supplementary_Data_S7.pdf]

Case Study I: TCGA-AN-A0XN

- 68-year-old woman
- Stage III breast cancer
- ER negative, PR positive, HER2 negative
- *luminal A* subtype according to PAM50
- T2, N2, M0

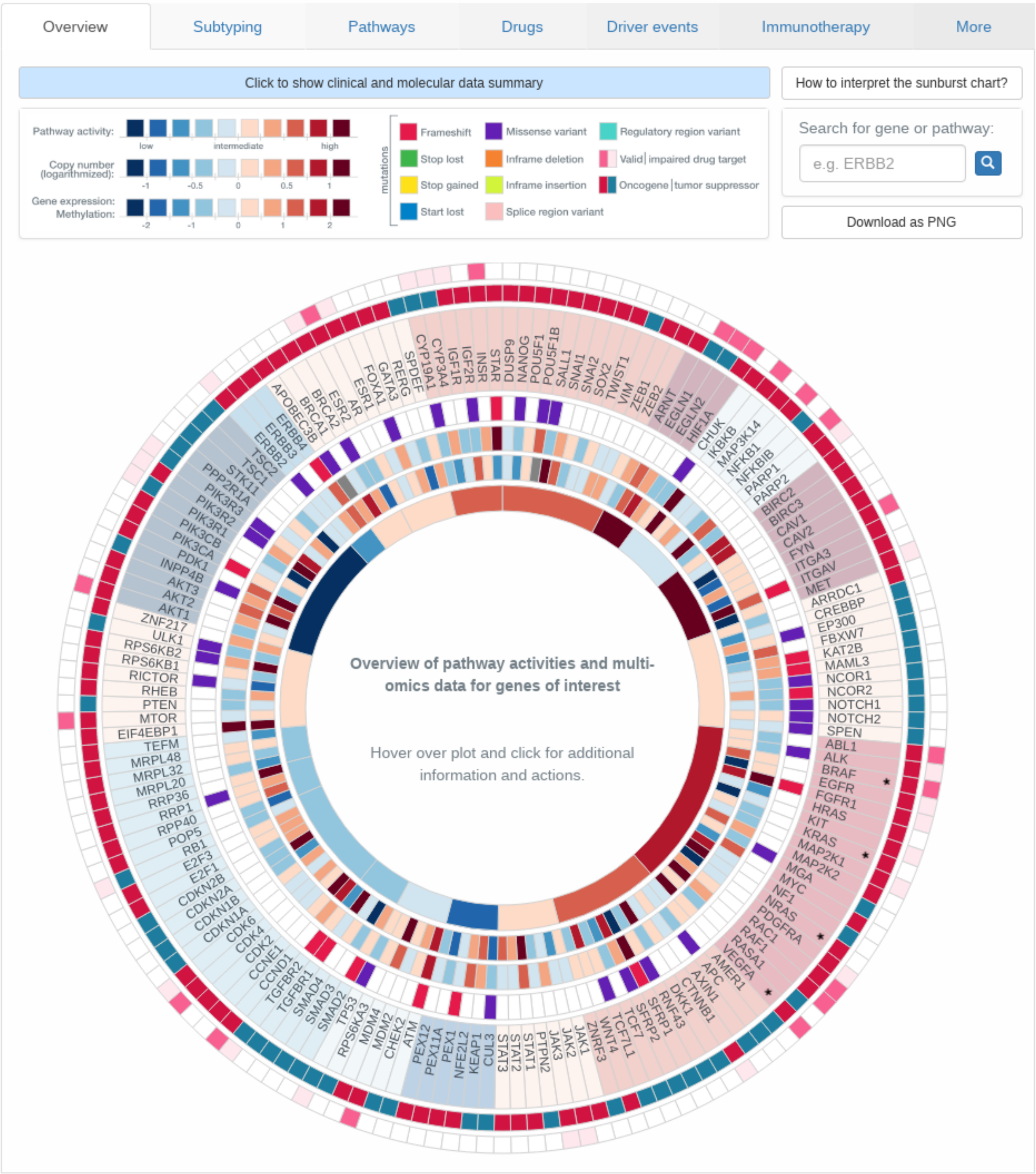

Figure 1: Sunburst chart overview for TCGA-AN-A0XN. Breast cancer-relevant driver genes and pathways are displayed in a circular manner. Genes are grouped according to the pathways they are most characteristic for. The plot is organized in rings, where the innermost ring displays pathway activities, the second 'inner' ring corresponds to gene expression. Depending on the data provided by the user, information on copy number alterations, and mutations is shown in the third and fourth ring respectively. Gene names are displayed in the next ring. The second most outer ring indicates whether the gene acts as an oncogene or tumor suppressor gene (TSG) for activating the corresponding pathway. The outermost ring contains indicators on whether or not the gene is a known drug target. Genes discussed in the manuscript are highlighted with an asterisk.

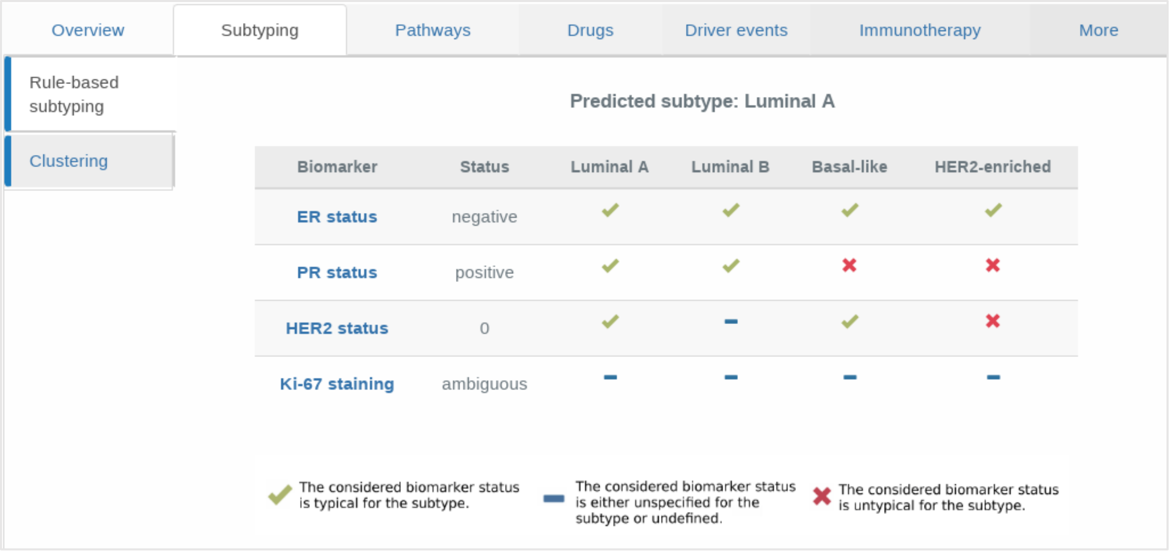

Figure 2: Rule-based subtyping for TCGA-AN-A0XN. Based on the hormone receptor and HER2 status of a tumor sample, as well as the observed growth rates, a classification into the four main breast cancer subtypes *Luminal A*, *Luminal B*, *Basal-like* and *HER2-enriched* can be performed.

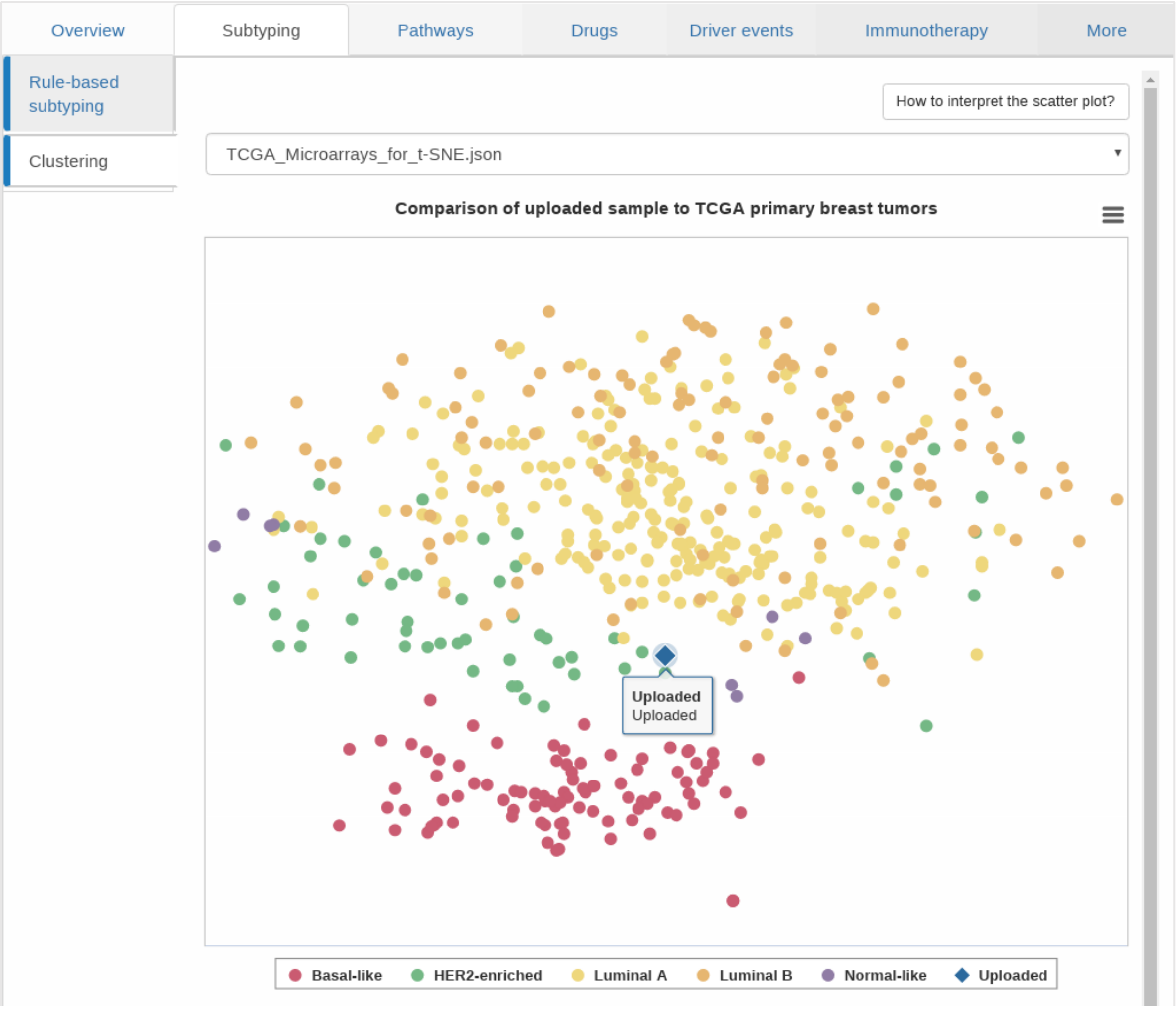

Figure 3: Clustering results for TCGA-AN-A0XN. The tumor sample of interest is clustered along with primary breast tumor samples from TCGA. The molecular subtypes of the TCGA samples are color-coded as indicated by the legend below the plot. The tumor sample under investigation is indicated by the blue diamond-shaped symbol.

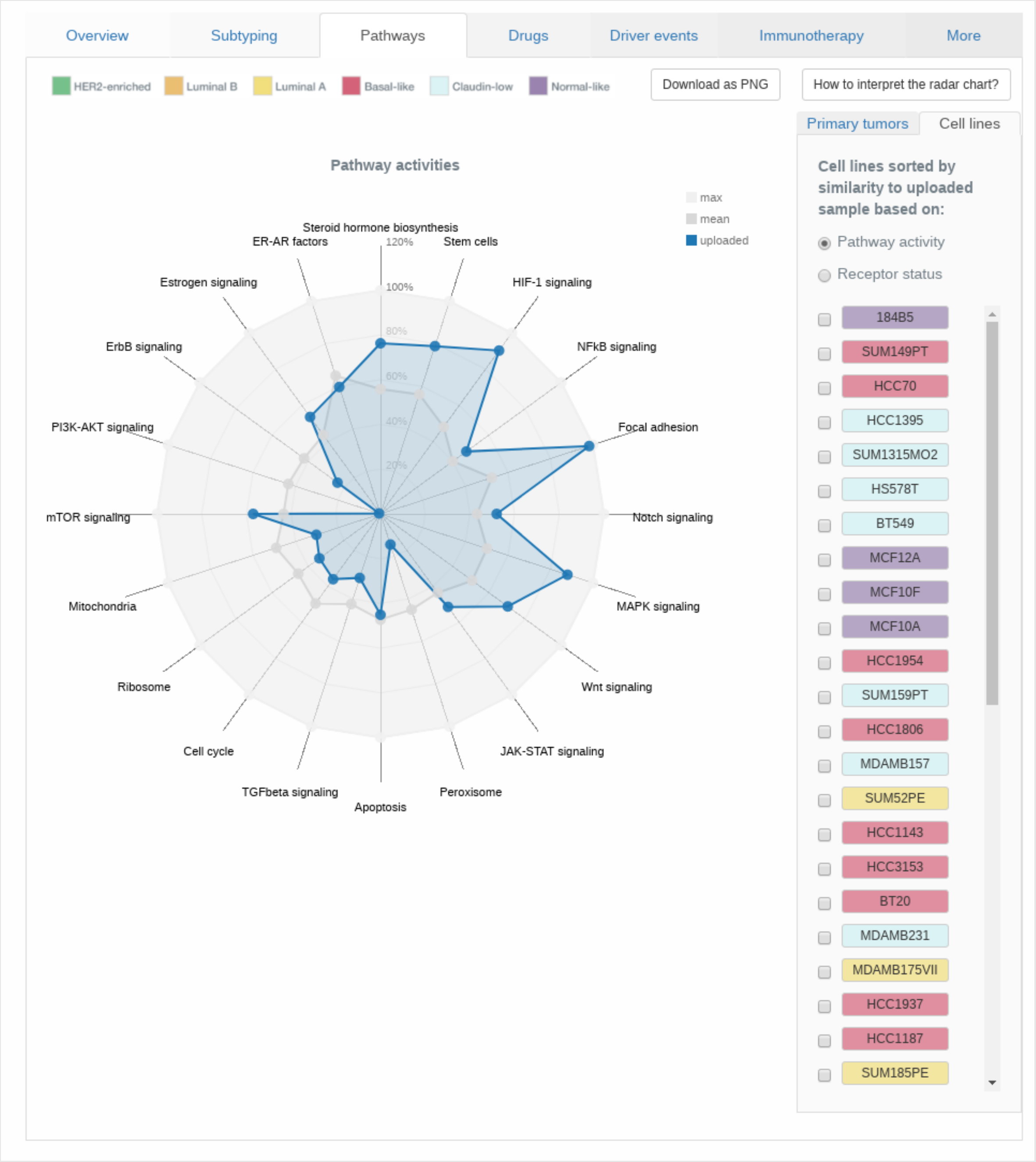

Figure 4: Radar chart of pathway activities for TCGA-AN-A0XN. The pathway activities of a set of 20 core breast cancer pathways for the user-provided tumor sample colored in blue. Reference samples from TCGA as well as breast cancer cell lines can be added to the visualization interactively. The molecular subtype of the respective reference samples is color-coded: basal-like - red, claudin-low - light blue, HER2-enriched - green, luminal A - yellow, luminal B – orange, normal-like - purple. Clicking on a reference sample's name yields additional clinical and pharmacological information, see Figure 5.

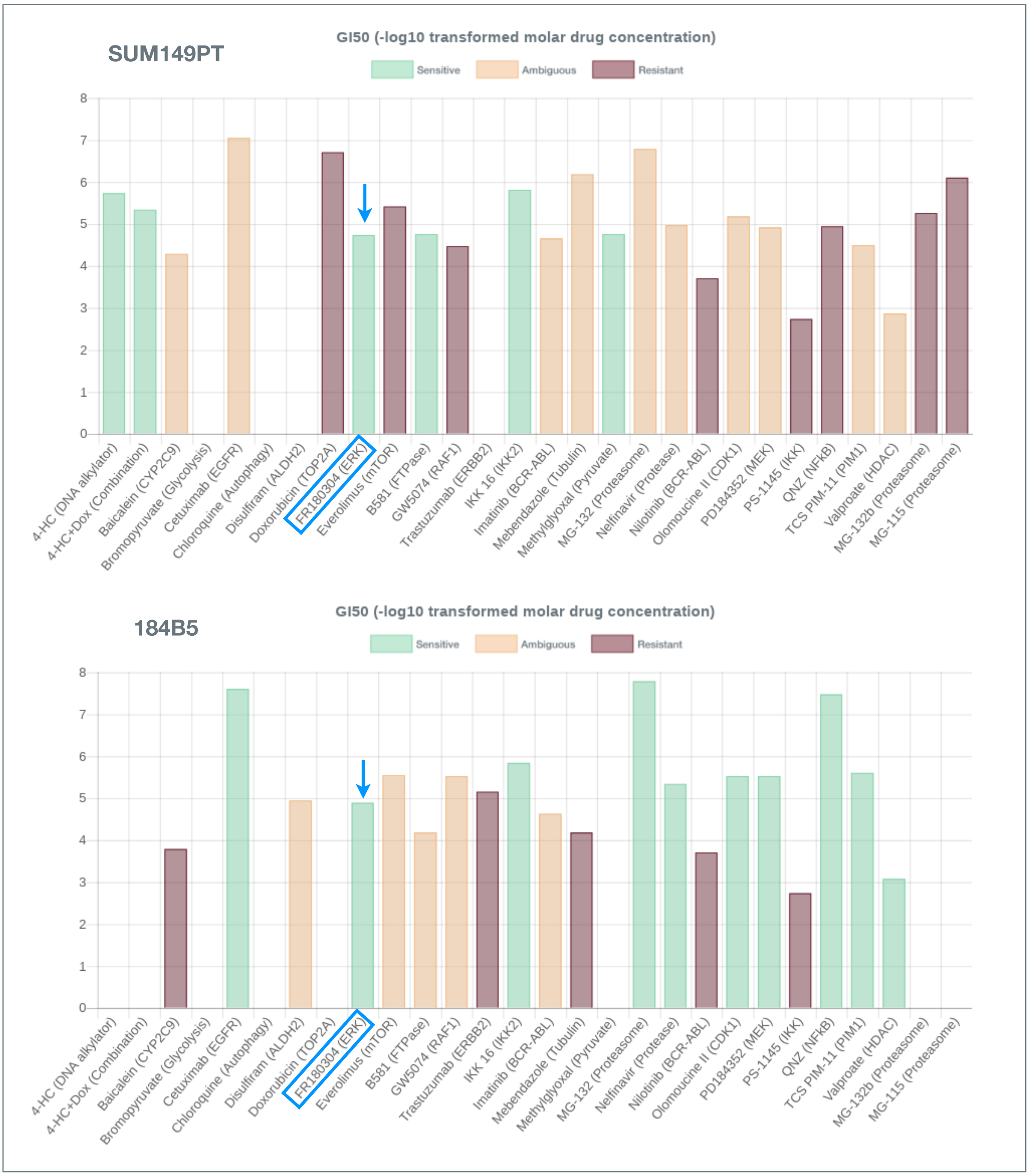

Figure 5: Drug sensitivity information for cell lines similar to TCGA-AN-A0XN. Similarity was assessed based on similarity of pathway activity patterns. The triple negative cell lines SUM149PT and 184B5 are most similar to the sample under investigation (cf. Figure 4). Especially, both cell lines were tested to be sensitive for ERK inhibitors (highlighted in blue) by (Heiser et al., 2012).

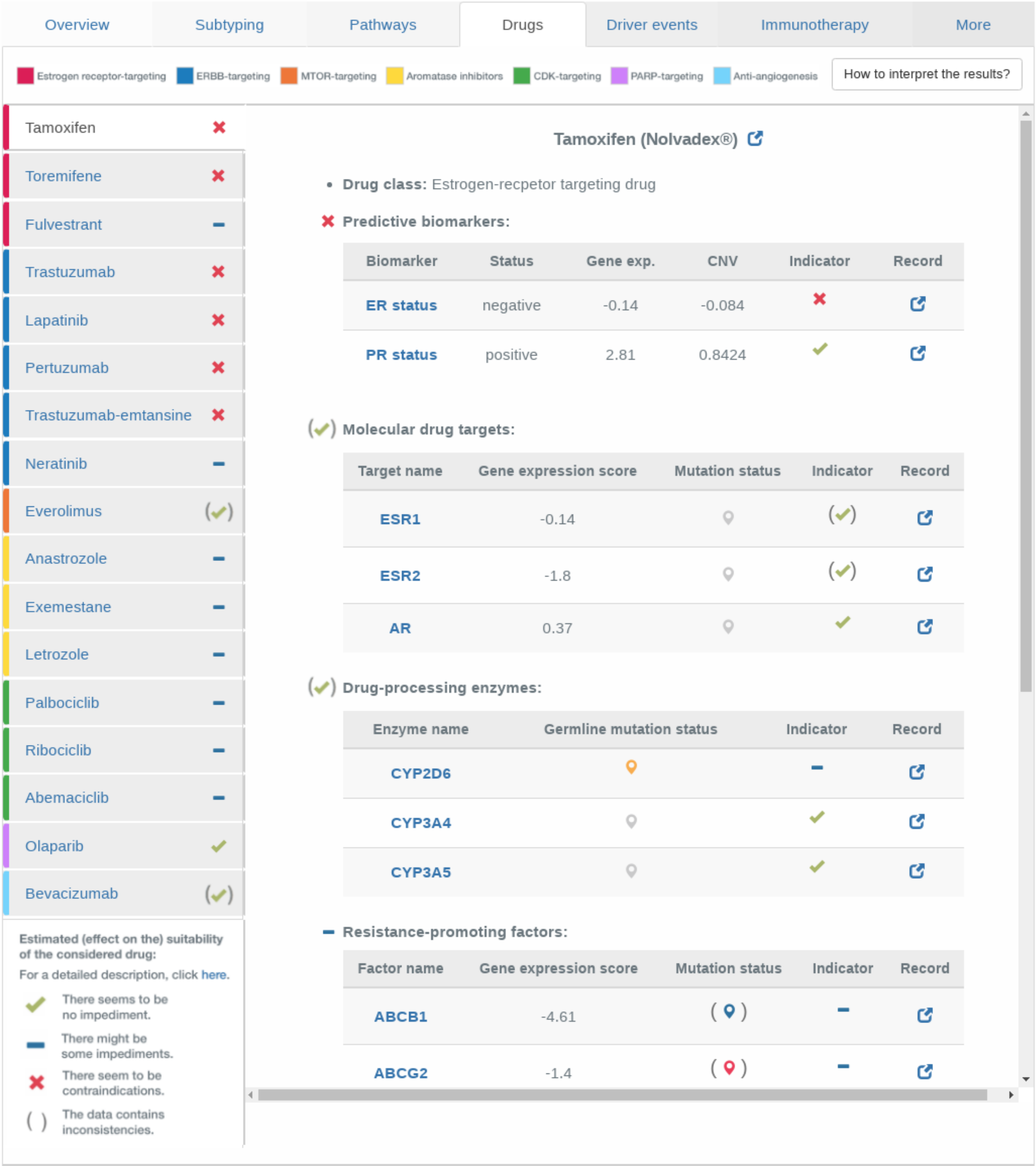

Figure 6: Assessment of recommended drugs for sample TCGA-AN-A0XN. For a set of 17 standard-of-care breast cancer drugs (left panel), various factors increasing or decreasing the efficacy of a drug are assessed. Clinical, genetic and molecular characteristics are listed with an indicator sign on whether they might decrease efficacy or even cause resistance to the treatment with the drug under consideration. All genes and pathways are linked to third-party resources where additional details can be found. Each entry also contains the link to a record or publication that describes the role of the corresponding gene with respect to the drug of interest.

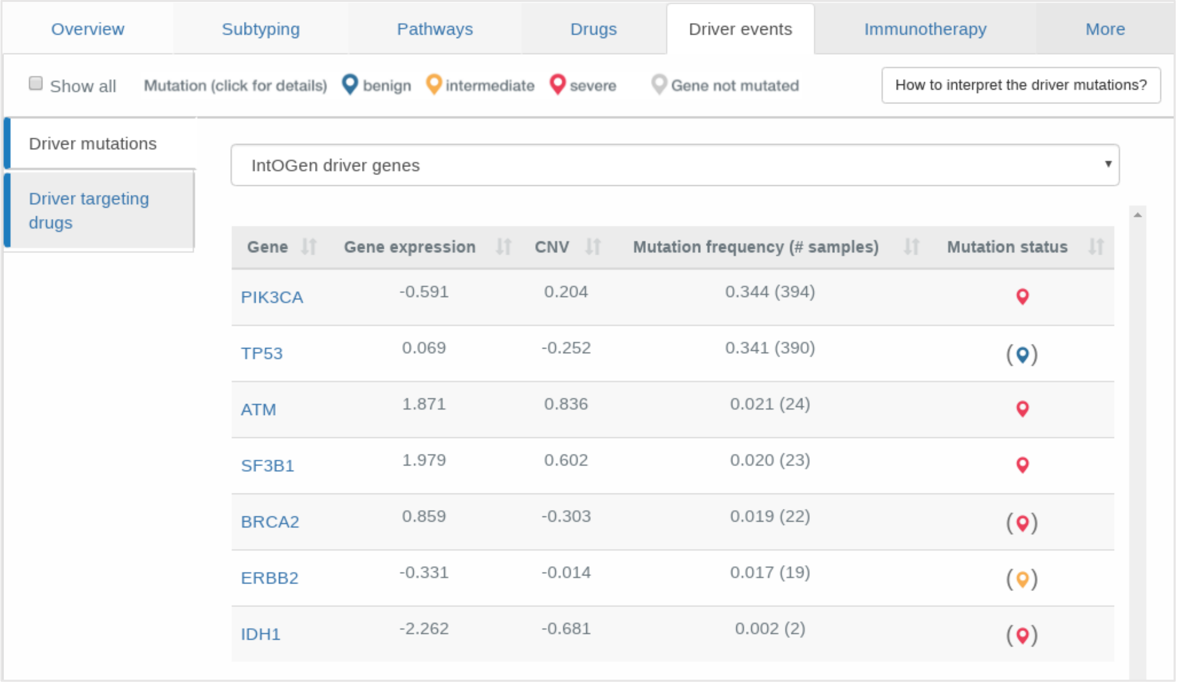

Gene	Gene expression	CNV	Mutation frequency (# samples)	Mutation status
PIK3CA	-0.591	0.204	0.344 (394)	severe
TP53	0.069	-0.252	0.341 (390)	(benign)
ATM	1.871	0.836	0.021 (24)	severe
SF3B1	1.979	0.602	0.020 (23)	severe
BRCA2	0.859	-0.303	0.019 (22)	(severe)
ERBB2	-0.331	-0.014	0.017 (19)	(intermediate)
IDH1	-2.262	-0.681	0.002 (2)	(severe)

Figure 7: Driver mutations in sample TCGA-AN-A0XN. Driver mutations contained in the sample under investigation. The color-code in the *Mutation* column indicates the severity of the mutation. Parentheses indicate a germline mutation. Clicking on the indicator symbol opens a modal with additional details on the specific contained mutation(s) and their predicted effect on protein functionality.

Overview | Subtyping | Pathways | Drugs | Driver events | Immunotherapy | More

Show all

How to interpret the driver mutations?

Driver mutations

Driver targeting drugs

Estimated suitability of the considered drug: ✓ There seems to be no impediment. — There might be some impediments. ✗ There seem to be contraindications.

Drug	Target	Alteration	Indicator
Ado-trastuzumab emtansine	HER2	HER2+	✗
Afatinib	EGFR/HER2	EGFR exon 19 deletion, L858R	✗
Brigatinib	ALK	ALK+	✓
Cetuximab	EGFR	KRAS wild type	✓
Dabrafenib	BRAF	BRAF V600E mutation	✗
Enasidenib	IDH2	IDH2 mutation	✗
Erlotinib	EGFR	EGFR exon 19 deletion, L858R	✗
Everolimus	mTOR	HR+, HER2-	✓
Gefitinib	EGFR	EGFR exon 19 deletion, L858R	✗
Lapatinib	HER2/ EGFR	HER2+	✗
Midostaurin	FLT3	FLT3+	✗
Neratinib	HER2	HER2+	✗
Olaparib	PARP	BRCA mutation	✓
Osimertinib	EGFR	EGFR T790M mutation	✗
Palbociclib	CDK4, CDK6	HR+, HER2-	✓
Panitumumab	EGFR	KRAS wild type	✓
Pembrolizumab	PD-1	PD-L1+	✓
Pertuzumab	HER2	HER2+	✗
Ribociclib	CDK4, CDK6	HR+, HER2-	✓

Figure 8: Assessment of driver targeting drugs for TCGA-AN-A0XN. This table contains driver-targeting drugs, i.e. those drugs that require the presence or absence of a specific mutation or other genomic alteration. The listed drugs are not necessarily approved for breast cancer and hence might be considered as off-label treatment options.

URL to interactive results:

https://clinomicstrail.bioinf.uni-sb.de/breast_cancer.html?title=ClinOmicsTrail&session=99c6f09a-b110-4a51-b3a3-b3e20f48a1bb

References

Heiser, L.M., Sadanandam, A., Kuo, W.-L., Benz, S.C., Goldstein, T.C., Ng, S., Gibb, W.J., Wang, N.J., Ziyad, S., Tong, F., Bayani, N., Hu, Z., Billig, J.I., Dueregger, A., Lewis, S., Jakkula, L., Korkola, J.E., Durinck, S., Pepin, F., Guan, Y., Purdom, E., Neuvial, P., Bengtsson, H., Wood, K.W., Smith, P.G., Vassilev, L.T., Hennessy, B.T.,

Greshock, J., Bachman, K.E., Hardwicke, M.A., Park, J.W., Marton, L.J., Wolf, D.M., Collisson, E.A., Neve, R.M., Mills, G.B., Speed, T.P., Feiler, H.S., Wooster, R.F., Haussler, D., Stuart, J.M., Gray, J.W., Spellman, P.T., 2012. Subtype and pathway specific responses to anticancer compounds in breast cancer. Proceedings of the National Academy of Sciences of the United States of America 109, 2724–2729. doi:10.1073/pnas.1018854108

Case Study II: TCGA-BH-A0DT

- 41-year-old woman
- Stage II breast cancer
- ER positive, PR positive, HER2 negative
- *luminal A* subtype according to PAM50
- T1, N1, M0

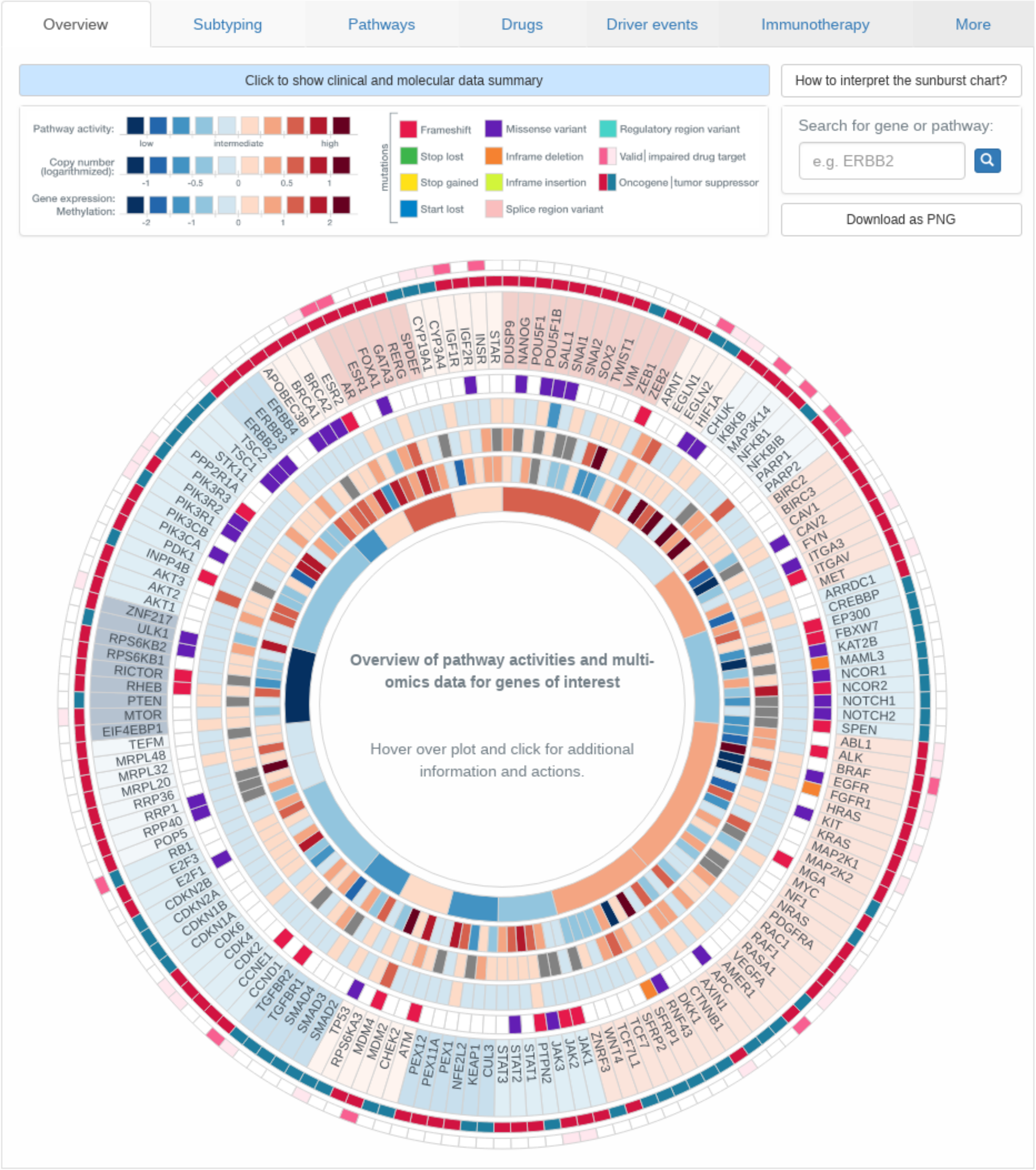

Figure 1: Sunburst chart overview for TCGA-BH-A0DT. Breast cancer-relevant driver genes and pathways are displayed in a circular manner. Genes are grouped according to the pathways they are most characteristic for. The plot is organized in rings, where the innermost ring displays pathway activities, the second 'inner' ring corresponds to gene expression. Depending on the data provided by the user, information on methylation scores, copy number alterations, and mutations is shown in the third, fourth, and fifth ring respectively. Gene names are displayed in the next ring. The second most outer ring indicates whether the gene acts as an oncogene or tumor suppressor gene (TSG) for activating the corresponding pathway. The outermost ring contains indicators on whether or not the gene is a known drug target.

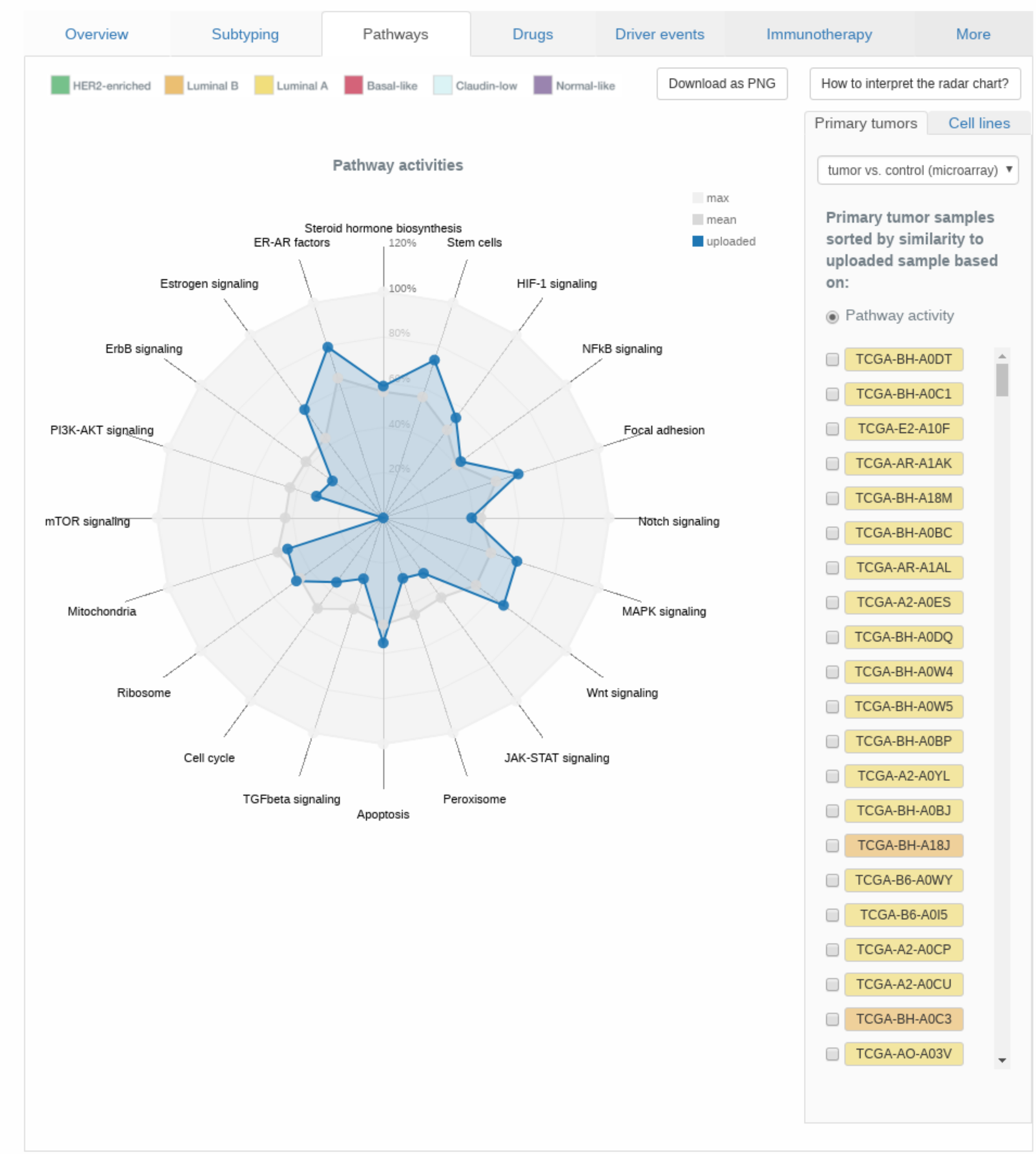

Figure 2: Radar chart of pathway activities for TCGA-BH-A0DT. The pathway activities of a set of 20 core breast cancer pathways for the user-provided tumor sample colored in blue. Reference samples from TCGA as well as breast cancer cell lines can be added to the visualization interactively. The molecular subtype of the respective reference samples is color-coded: basal-like - red, claudin-low - light blue, HER2-enriched - green, luminal A - yellow, luminal B – orange, normal-like - purple.

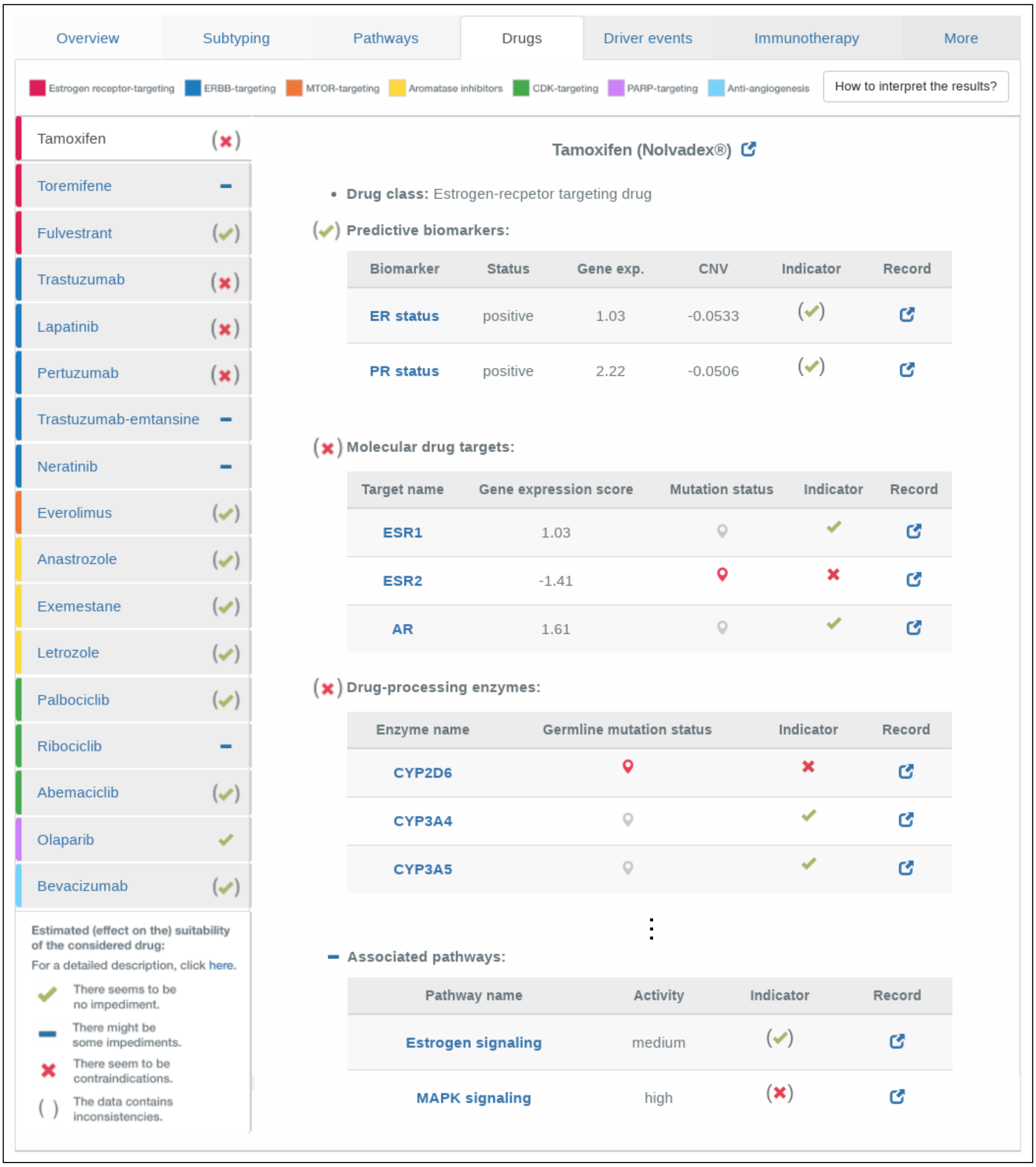

Figure 3: Assessment of tamoxifen for TCGA-BH-A0DT. For a set of 17 standard-of-care breast cancer drugs (left panel), various factors increasing or decreasing the efficacy of a drug are assessed. Clinical, genetic and molecular characteristics are listed with an indicator sign on whether they might decrease efficacy or even cause resistance to the treatment with the drug under consideration. All genes and pathways are linked to third-party resources where additional details can be found. Each entry also contains the link to a record or publication that describes the role of the corresponding gene with respect to the drug of interest. Clicking on the indicator symbol in the *Germline mutation status* column for CYP2D6 will open a window with additional details, cf. Figure 4.

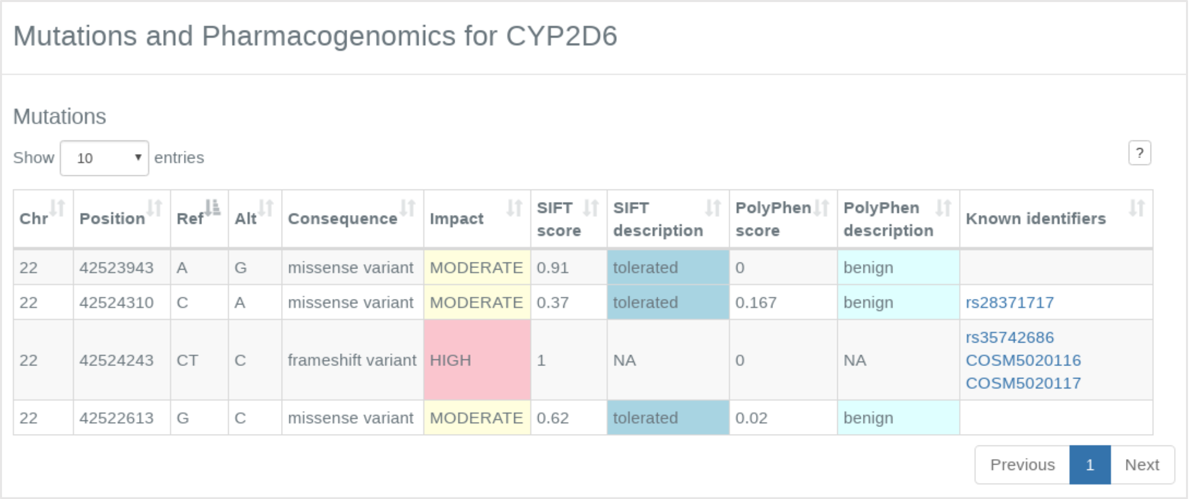

Mutations and Pharmacogenomics for CYP2D6

Mutations

Show 10 entries ?

Chr	Position	Ref	Alt	Consequence	Impact	SIFT score	SIFT description	PolyPhen score	PolyPhen description	Known identifiers
22	42523943	A	G	missense variant	MODERATE	0.91	tolerated	0	benign	
22	42524310	C	A	missense variant	MODERATE	0.37	tolerated	0.167	benign	rs28371717
22	42524243	CT	C	frameshift variant	HIGH	1	NA	0	NA	rs35742686 COSM5020116 COSM5020117
22	42522613	G	C	missense variant	MODERATE	0.62	tolerated	0.02	benign	

Previous 1 Next

Figure 4: Detailed view on mutations in gene CYP2D6. Clicking on the indicator symbol of a mutation opens a modal with additional details on the specific mutations contained in the gene of interest, as well as an estimation of the mutations severities based on VeP Impact, SIFT and PolyPhen.

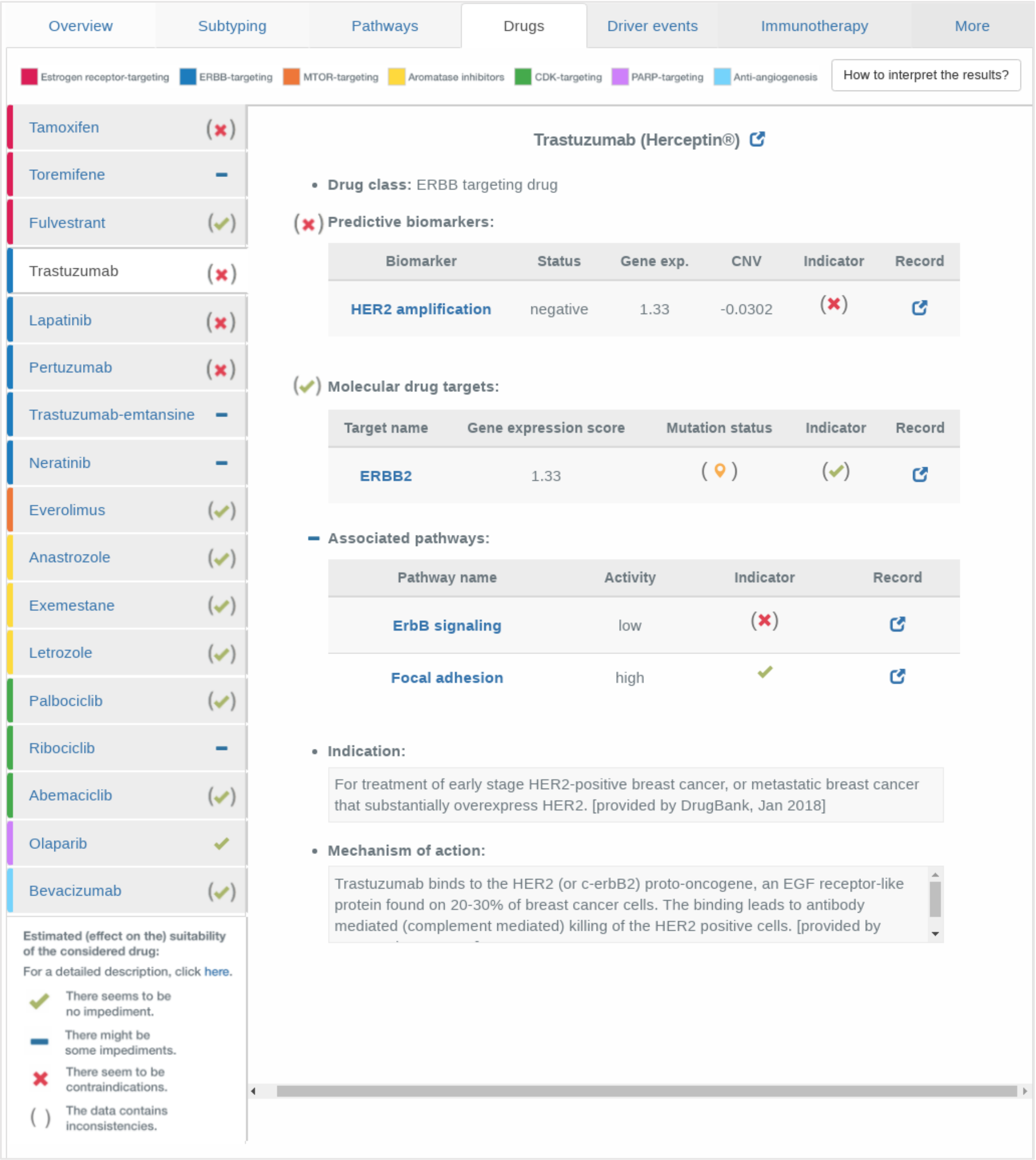

Figure 5: Assessment of trastuzumab for TCGA-BH-A0DT. For a set of 17 standard-of-care breast cancer drugs (left panel), various factors increasing or decreasing the efficacy of a drug are assessed. Clinical, genetic and molecular characteristics are listed with an indicator sign on whether they might decrease efficacy or even cause resistance to the treatment with the drug under consideration. All genes and pathways are linked to third-party resources where additional details can be found. Each entry also contains the link to a record or publication that describes the role of the corresponding gene with respect to the drug of interest.

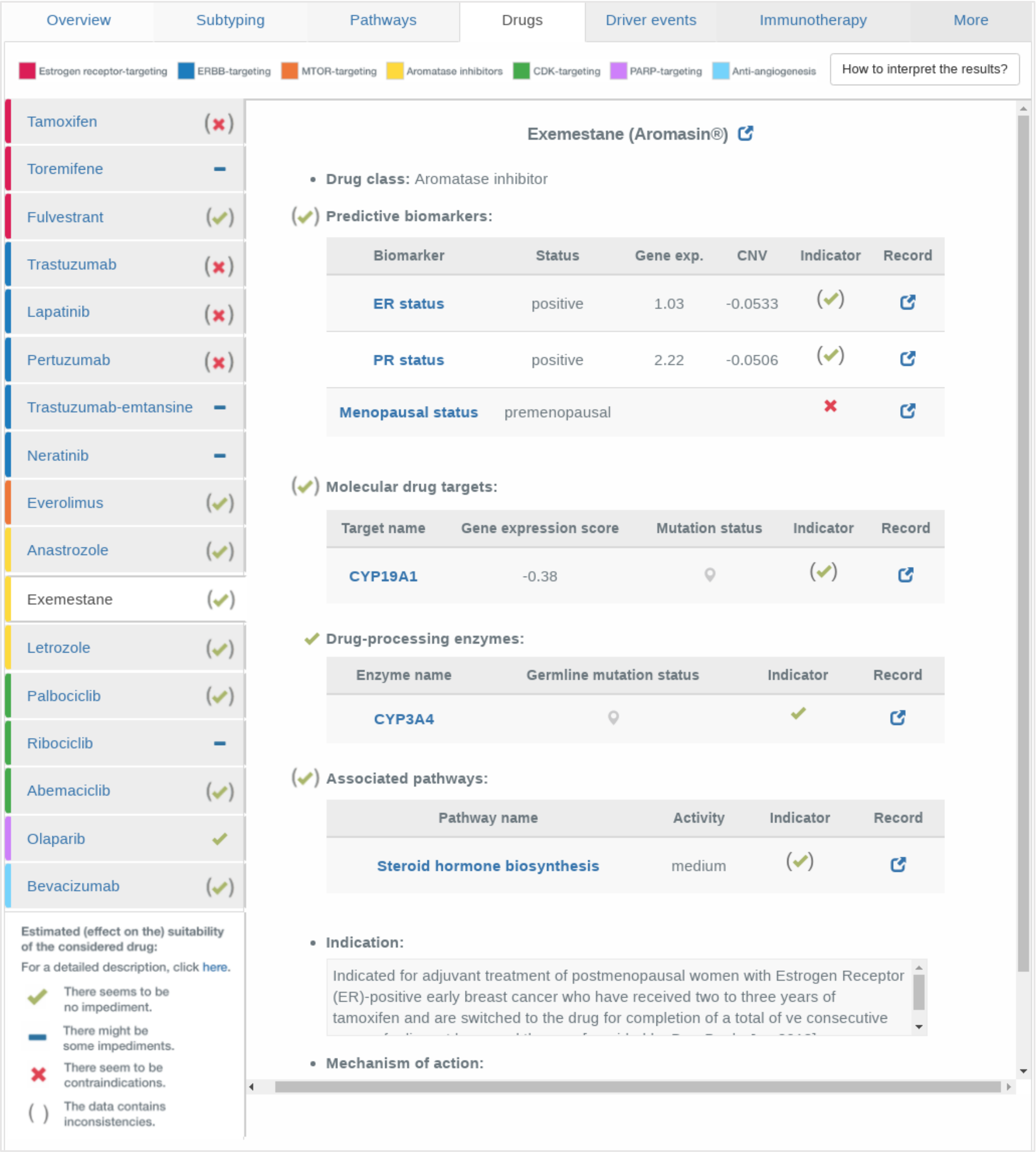

Figure 6: Assessment of aromatase inhibitor exemestane for TCGA-BH-A0DT. For a set of 17 standard-of-care breast cancer drugs (left panel), various factors increasing or decreasing the efficacy of a drug are assessed. Clinical, genetic and molecular characteristics are listed with an indicator sign on whether they might decrease efficacy or even cause resistance to the treatment with the drug under consideration. All genes and pathways are linked to third-party resources where additional details can be found. Each entry also contains the link to a record or publication that describes the role of the corresponding gene with respect to the drug of interest.

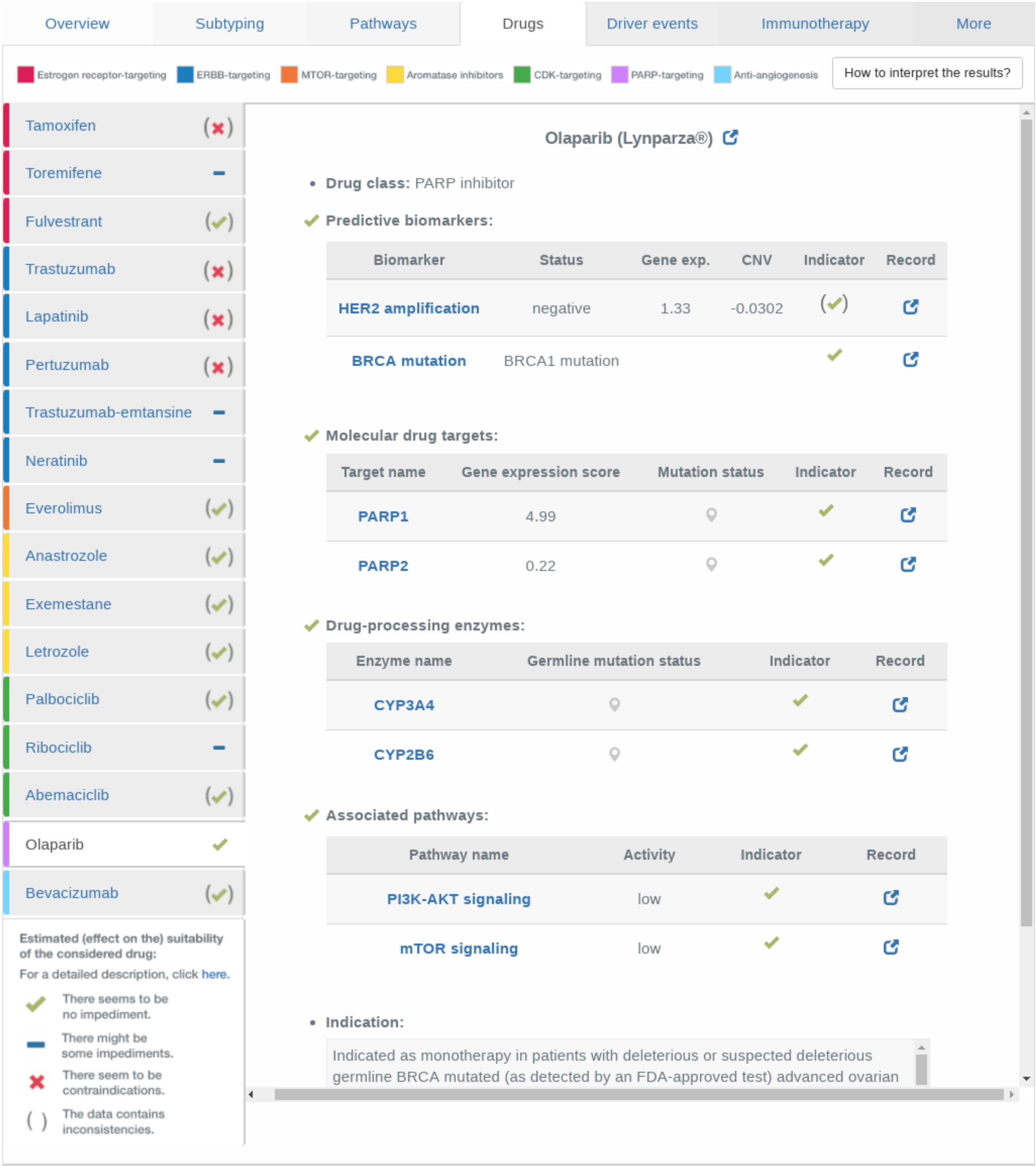

Figure 7: Assessment of olaparib for TCGA-BH-A0DT. For a set of 17 standard-of-care breast cancer drugs (left panel), various factors increasing or decreasing the efficacy of a drug are assessed. Clinical, genetic and molecular characteristics are listed with an indicator sign on whether they might decrease efficacy or even cause resistance to the treatment with the drug under consideration. All genes and pathways are linked to third-party resources where additional details can be found. Each entry also contains the link to a record or publication that describes the role of the corresponding gene with respect to the drug of interest.

URL to interactive results:

https://clinomicstrail.bioinf.uni-sb.de/breast_cancer.html?title=ClinOmicsTrail&session=50d40dbd-c31e-404d-8a7e-ac796bbd5d7b

Case Study III: TCGA-A2-A0T2

- 66-year-old woman
- Stage IV breast cancer
- ER negative, PR negative, HER2 negative
- *Basal-like* subtype according to PAM50
- T3, N3, M1

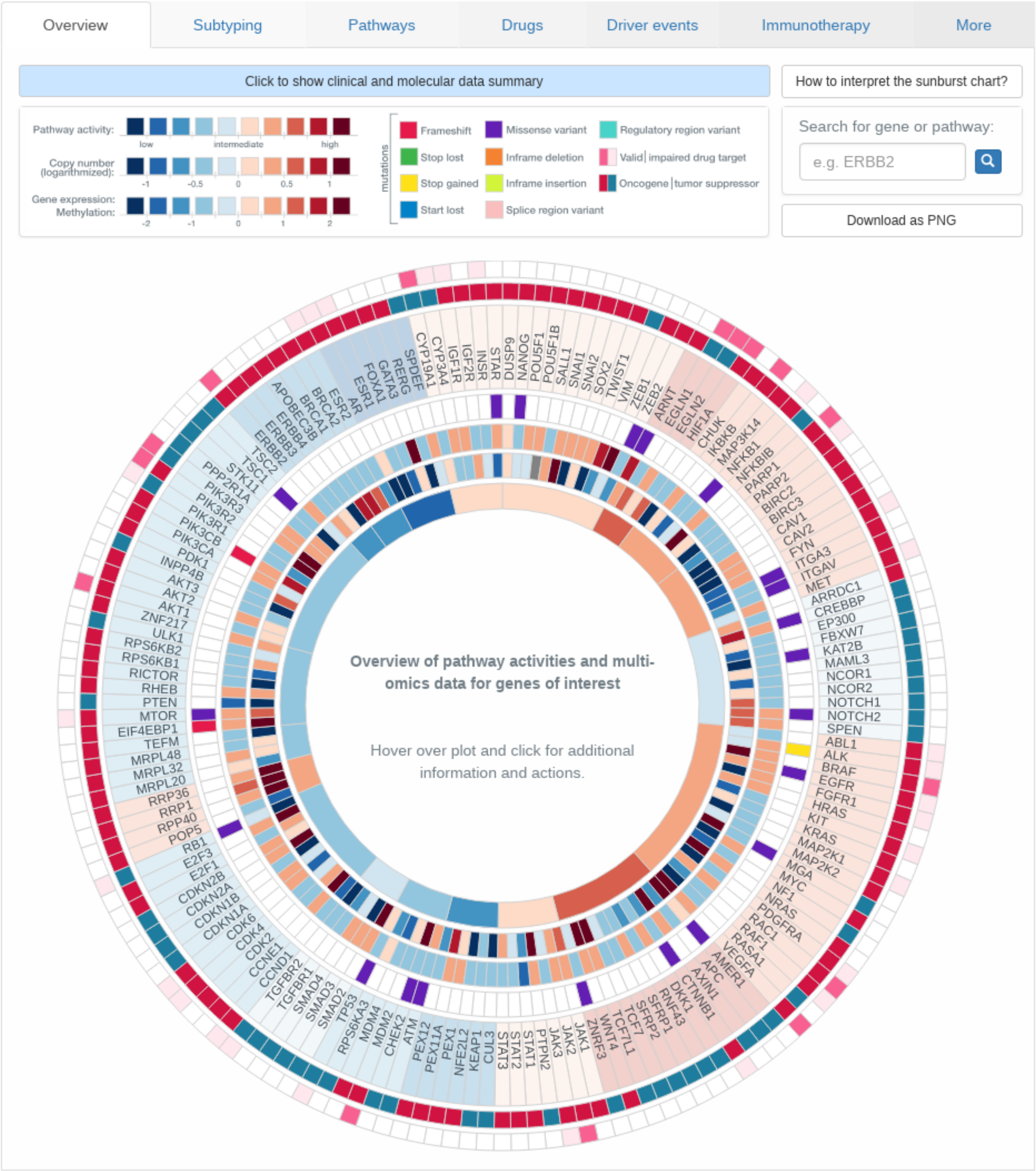

Figure 1: Sunburst chart overview for TCGA-A2-A0T2. Breast cancer-relevant driver genes and pathways are displayed in a circular manner. Genes are grouped according to the pathways they are most characteristic for. The plot is organized in rings, where the innermost ring displays pathway activities, the second 'inner' ring corresponds to gene expression. Depending on the data provided by the user, information on copy number alterations and mutations is shown in the third and fourth ring respectively. Gene names are displayed in the next ring. The second most outer ring indicates whether the gene acts as an oncogene or tumor suppressor gene (TSG) for activating the corresponding pathway. The outermost ring contains indicators on whether or not the gene is a known drug target.

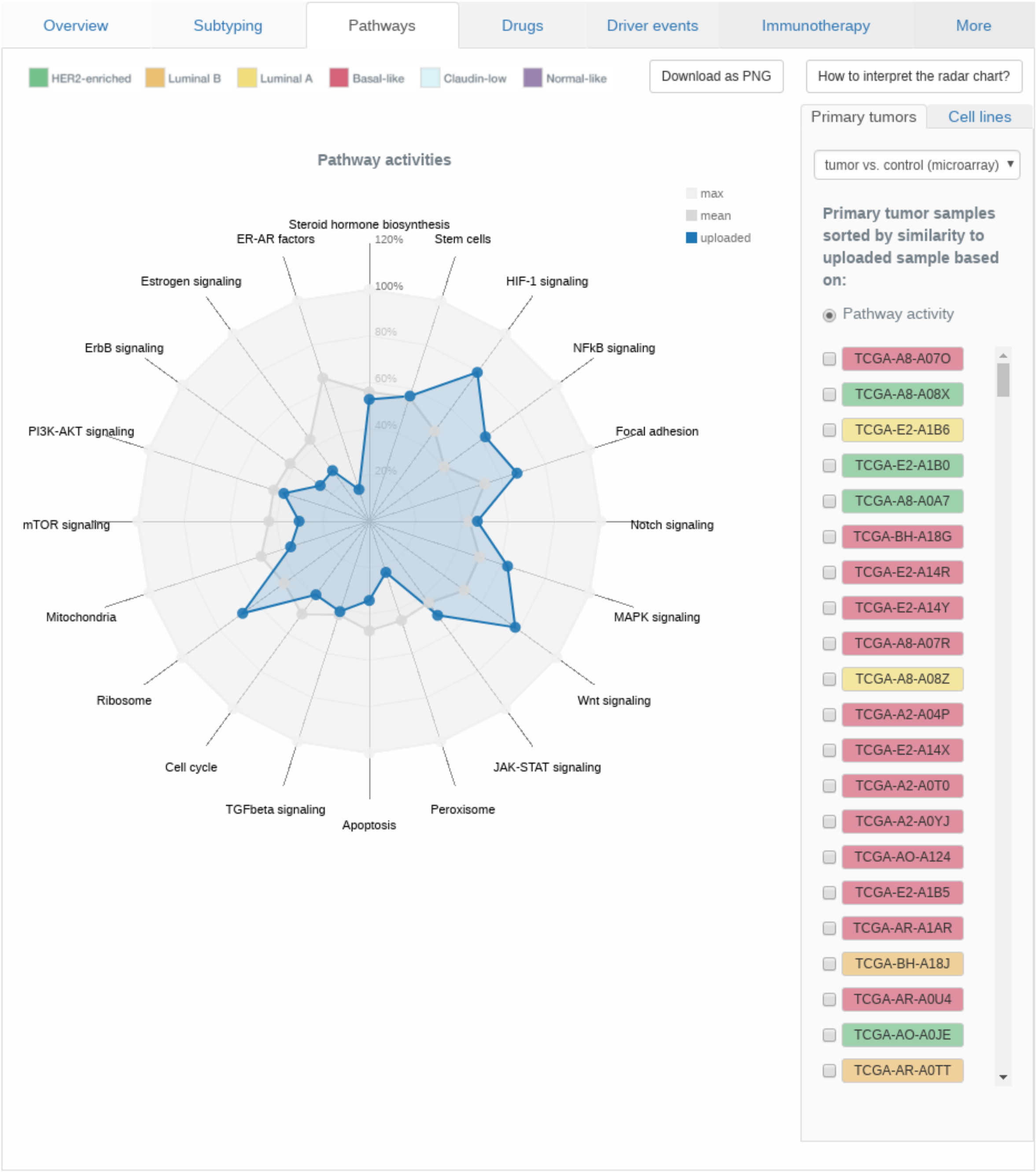

Figure 2: Radar chart of pathway activities for TCGA-A2-A0T2. The pathway activities of a set of 20 core breast cancer pathways for the user-provided tumor sample colored in blue. Reference samples from TCGA as well as breast cancer cell lines can be added to the visualization interactively. The molecular subtype of the respective reference samples is color-coded: basal-like - red, claudin-low - light blue, HER2-enriched - green, luminal A - yellow, luminal B – orange, normal-like - purple.

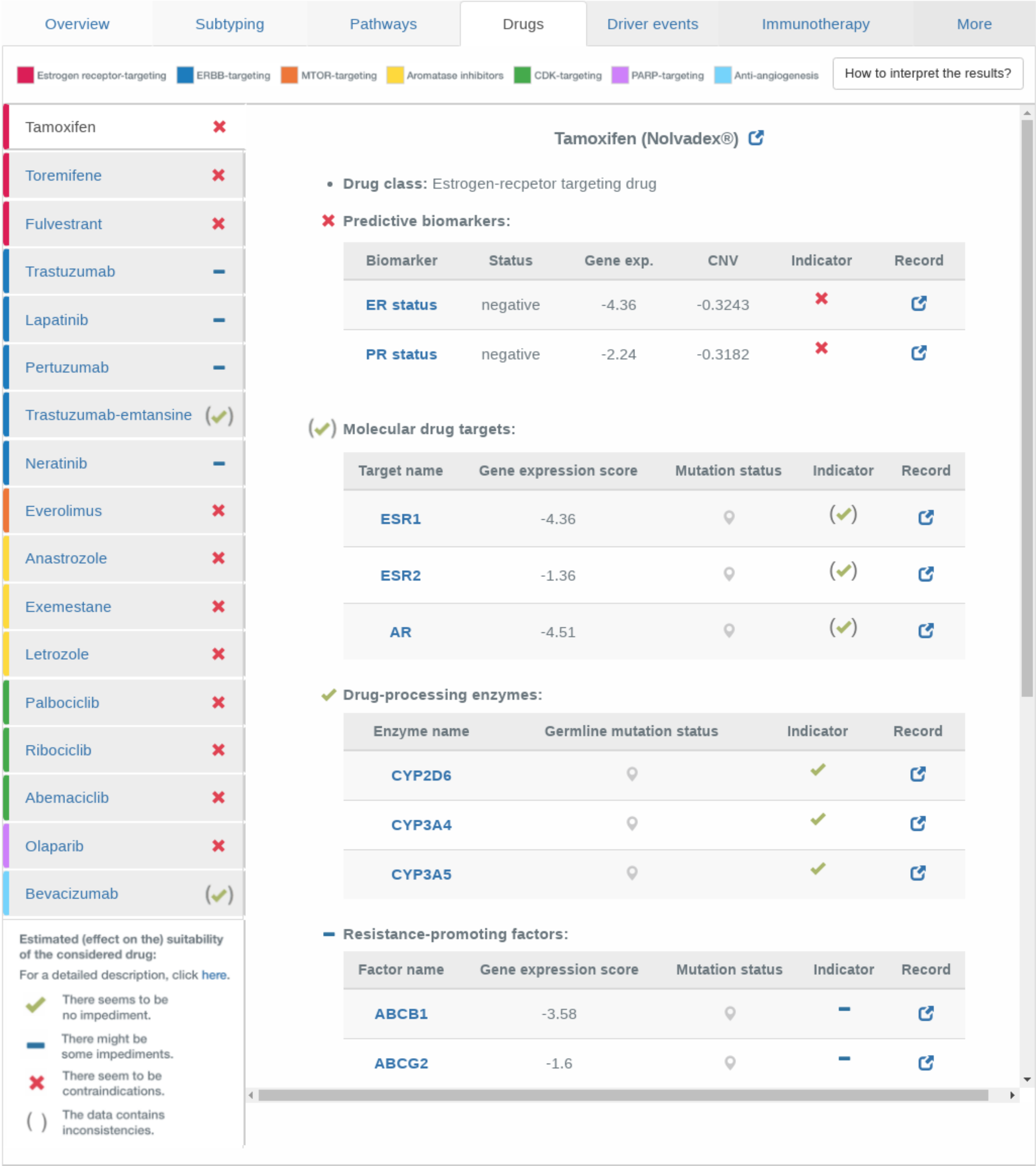

Figure 3: Drug assessment for TCGA-A2-A0T2. For a set of 17 standard-of-care breast cancer drugs (left panel), various factors increasing or decreasing the efficacy of a drug are assessed. Clinical, genetic and molecular characteristics are listed with an indicator sign on whether they might decrease efficacy or even cause resistance to the treatment with the drug under consideration. All genes and pathways are linked to third-party resources where additional details can be found. Each entry also contains the link to a record or publication that describes the role of the corresponding gene with respect to the drug of interest.

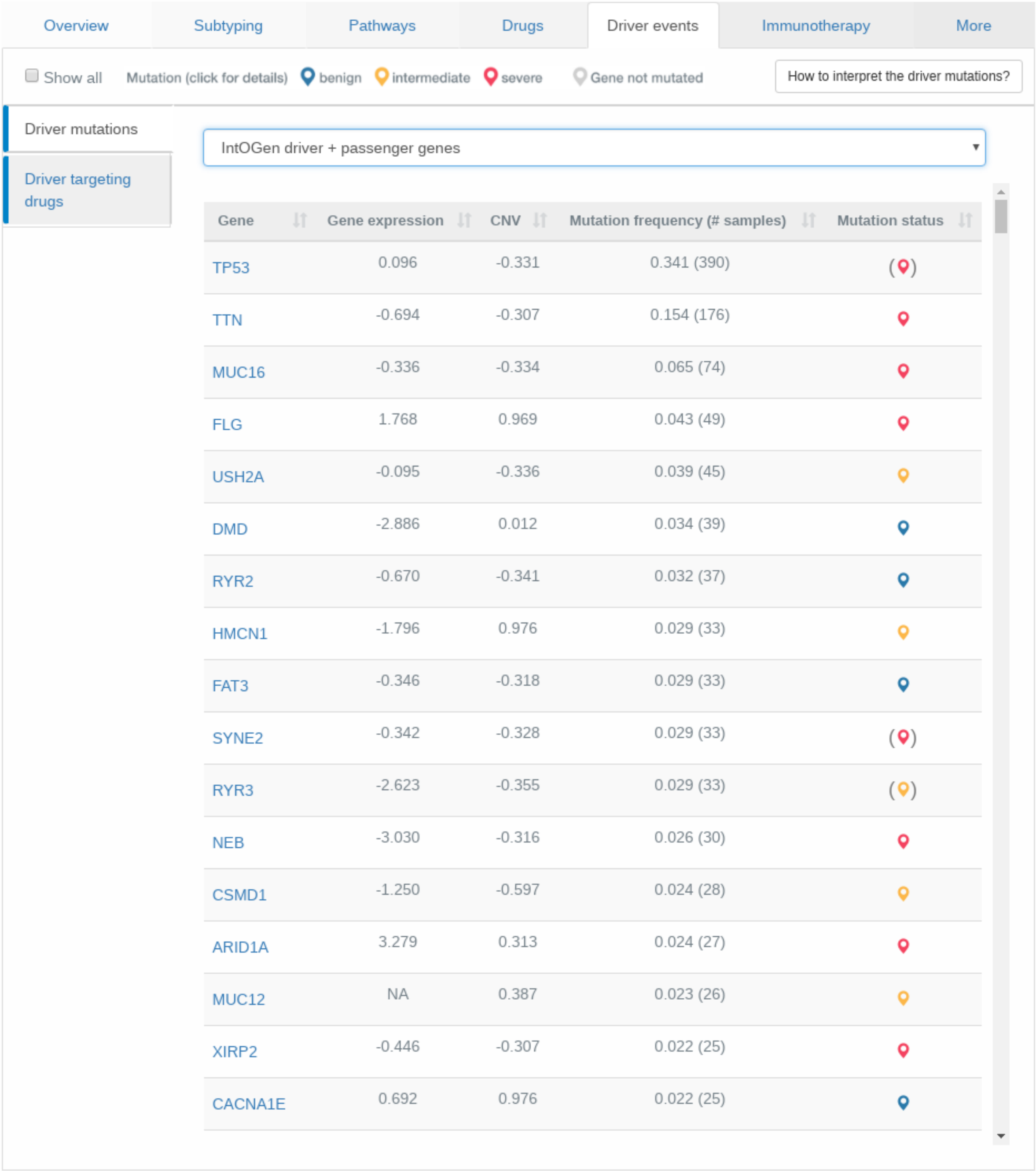

Figure 4: Driver (and passenger) mutations in TCGA-A2-A0T2. The table contains genes commonly mutated in breast cancer samples that are also mutated in the sample under consideration. The mutations are sorted by decreasing frequency. The color-code in the *Mutation status* column indicates the severity of the contained mutations. Clicking on the respective symbol will open a modal with additional details on the contained mutations and their putative effect on protein functionality.

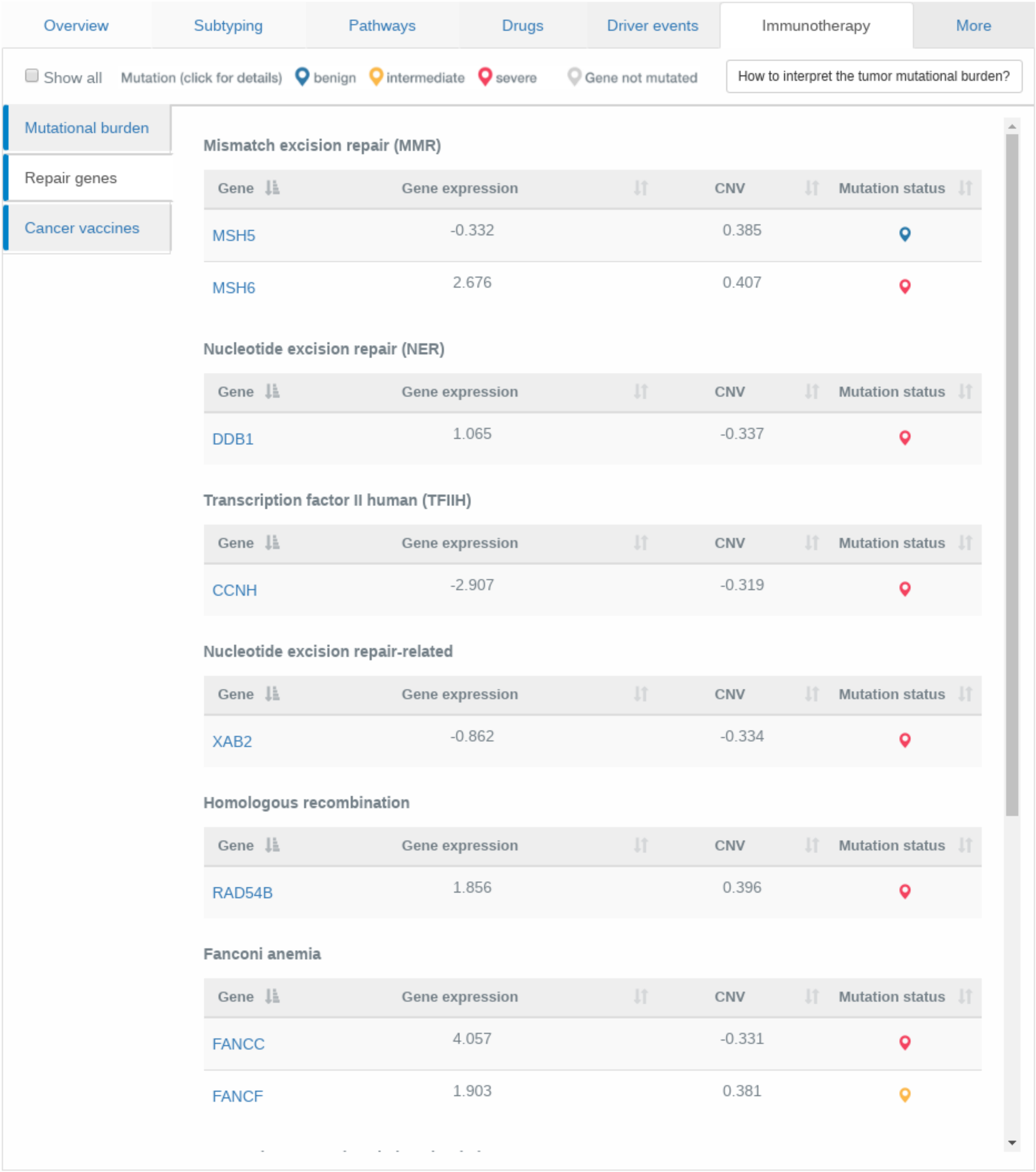

Figure 5: Impaired repair genes in TCGA-A2-A0T2. The table contains genes involved in a variety of repair processes that are impaired (i.e. mutated) in the sample under investigation. The color-code in the *Mutation status* column indicates the severity of the contained mutations. Clicking on the respective symbol will open a modal with additional details on the contained mutations and their putative effect on protein functionality.

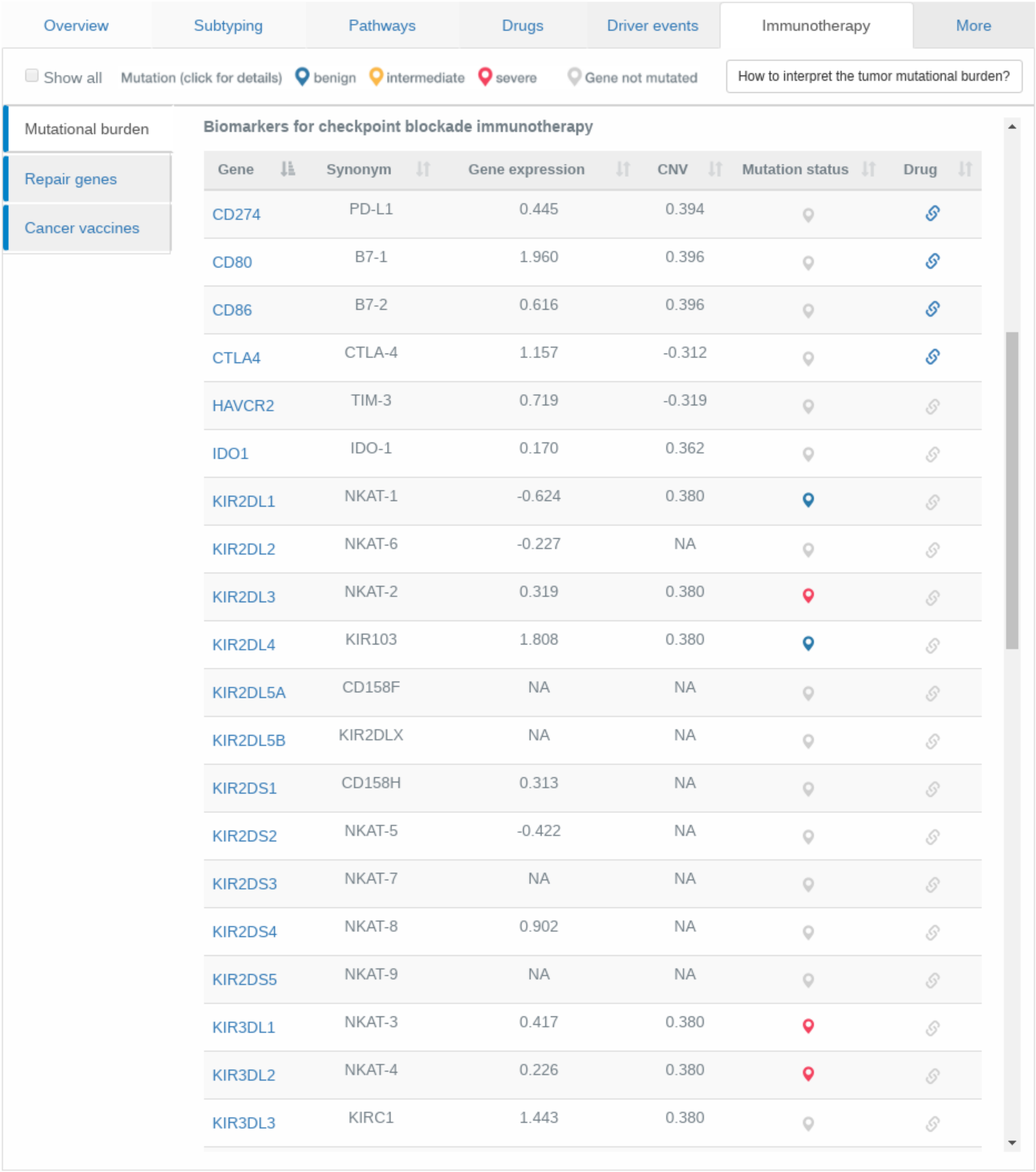

Gene	Synonym	Gene expression	CNV	Mutation status	Drug
CD274	PD-L1	0.445	0.394		
CD80	B7-1	1.960	0.396		
CD86	B7-2	0.616	0.396		
CTLA4	CTLA-4	1.157	-0.312		
HAVCR2	TIM-3	0.719	-0.319		
IDO1	IDO-1	0.170	0.362		
KIR2DL1	NKAT-1	-0.624	0.380		
KIR2DL2	NKAT-6	-0.227	NA		
KIR2DL3	NKAT-2	0.319	0.380		
KIR2DL4	KIR103	1.808	0.380		
KIR2DL5A	CD158F	NA	NA		
KIR2DL5B	KIR2DLX	NA	NA		
KIR2DS1	CD158H	0.313	NA		
KIR2DS2	NKAT-5	-0.422	NA		
KIR2DS3	NKAT-7	NA	NA		
KIR2DS4	NKAT-8	0.902	NA		
KIR2DS5	NKAT-9	NA	NA		
KIR3DL1	NKAT-3	0.417	0.380		
KIR3DL2	NKAT-4	0.226	0.380		
KIR3DL3	KIRC1	1.443	0.380		

Figure 6: Biomarkers for checkpoint inhibition in TCGA-A2-A0T2. The table contains biomarkers for checkpoint blockade immunotherapy. In cases the listed genes are the molecular targets of immunotherapeutic drugs, the indicator mark in the *Drug* column is colored in blue. Clicking on this mark yields additional information on the targeting drugs.

Show 10 entries | Strong binder | Weak binder | No binder | Search:

Sequence	A*02:01	A*24:02	B*15:17	B*40:01	C*07:01	Antigen ID
LWTGWVCCV	0.204	0.193	0.066	0.049	0.124	CENPL
LYVVTEAGE	0.024	0.045	0.034	0.054	0.016	GSDMC
MAFLAQKAI	0.167	0.052	0.691	0.089	0.254	TRIM24
MVFVAGQGV	0.426	0.035	0.674	0.125	0.307	SH3BP5L
NGGALGHDE	0.018	0.01	0.013	0.036	0.022	ABTB2
NISVSVPQP	0.044	0.019	0.028	0.048	0.022	UBAP2
NKAPQPMKG	0.016	0.016	0.008	0.033	0.035	SUMF2
NKAPQPMKP	0.016	0.02	0.009	0.041	0.037	SUMF2
NKAPQPMKS	0.017	0.012	0.008	0.033	0.038	SUMF2
NKAPQPMKV	0.063	0.033	0.014	0.049	0.107	SUMF2

Showing 181 to 190 of 326 entries

1 … 18 19 20 … 33

Figure 7: Neoepitope prediction for TCGA-A2-A0T2. The table contains all peptide sequences of length 9 that were predicted by NetMHC for significantly upregulated mutated genes. For each HLA, their binding affinities to the respective neoepitopes are listed in their respective columns. Affinity scores can be interpreted as 1-log50k(IC50). Yellow cells indicate weak binders, green cells strong binders. The last column contains the gene symbol of the antigen-providing gene in the tumor.

URL to interactive results:

https://clinomicstrail.bioinf.uni-sb.de/breast_cancer.html?title=ClinOmicsTrail&session=393a44fd-f15d-4e31-a974-3054bb386f5b

For neoepitope prediction results:

https://clinomicstrail.bioinf.uni-sb.de/epitope_results.html?resource=2242&epitopeLength=9&epitopePredictionMethod=netmhc&vcf&session=393a44fd-f15d-4e31-a974-3054bb386f5b
