# Supplementary material for: ClinOmicsTrailbc: a visual analytics tool for breast cancer treatment stratification
Source: Bioinformatics. 2019 Apr 30;35(24):5171–81. doi: 10.1093/bioinformatics/btz302 (PMC6954665; doi:10.1093/bioinformatics/btz302)
Supplement: btz302_Supplementary_Data [file btz302_supplementary_data.zip › btz302-Suppl_data/Supplementary_Data_S9.pdf]

Neoepitope Prediction for TCGA-A2-A0T2

Besides checkpoint blockade, personalized cancer vaccines are another promising approach to cancer immunotherapy[1,2]. Cancer vaccines target overexpressed or altered proteins and HLA presented peptide sequences (neoepitopes) that resulted from somatic mutations uniquely characterizing the patient's tumor. They are used to prime T cells to recognize these characterizing antigens and destroy the presenting tumor cells. As the neoepitopes are dependent on both, the patient's tumor mutations and HLA genotype, cancer vaccines have to be individually designed. To this end, ClinOmicsTrail[bc] offers functionalities to predict potential neoepitope vaccine targets based on the identified somatic mutations and HLA genotype of a patient using the immunoinformatic toolbox ImmunoNodes[3]. It provides various classes of epitope prediction methods to compute (neo-)epitopes and to assess their affinity to the patient's HLA genotype.

We here identified the HLA type for sample TCGA-A2-A0T2 using the HLA genotyping algorithm OptiType[4] based on raw tumor sequencing data as obtained from TCGA[5]. OptiType predicted the sample to be of the following genotype: A*02:01, A*24:02, B*15:17, B*40:01, C*07:01, C*03:04.

Within ClinOmicsTrail[bc], we selected the option *Consider only significantly up-regulated proteins* in the 'Cancer vaccines' tab of the 'Immunotherapy' view and performed a neoepitope prediction for peptides of length 9 using NetMHC[6]. Table 1 lists all neoepitopes that were predicted to bind to at least one of the sample's HLA proteins.

Sequence	A*02:01	A*24:02	B*15:17	B*40:01	C*07:01	Antigen
AVWALCYGY	0.17	0.026	0.46	0.076	0.066	ZFP42
FKTTRIIFY	0.084	0.03	0.061	0.061	0.44	AGO2
FLLDMVYRS	0.771	0.05	0.031	0.042	0.074	DOPEY2
FSFGPQPYY	0.104	0.03	0.931	0.062	0.436	ROR1
FSPYNGGAL	0.075	0.059	0.519	0.137	0.16	ABTB2
GELINNTVL	0.057	0.027	0.054	0.841	0.052	GSDMC
GTSPSLIFL	0.437	0.058	0.514	0.198	0.239	SLC13A4
IGTPPSLIF	0.048	0.247	0.533	0.067	0.067	SLC13A4
IGTPTSLIF	0.06	0.301	0.535	0.077	0.087	SLC13A4
LAEVLAFLL	0.197	0.098	0.462	0.146	0.076	DOPEY2
LAFLLDMVY	0.118	0.032	0.741	0.076	0.096	DOPEY2
LAQKAIKQW	0.035	0.091	0.677	0.046	0.054	TRIM24
LLAEVLAFL	0.848	0.144	0.153	0.114	0.139	DOPEY2
MAFLAQKAI	0.167	0.052	0.691	0.089	0.254	TRIM24
MVFVAGQGV	0.426	0.035	0.674	0.125	0.307	SH3BP5L
QAACPPAIF	0.051	0.153	0.519	0.101	0.021	FANCC
RFKTTRIIF	0.048	0.44	0.101	0.096	0.087	AGO2
RVILAKRLY	0.055	0.035	0.649	0.074	0.265	AXDND1
STRFKTTRI	0.066	0.081	0.634	0.062	0.149	AGO2
TIIGTPPSL	0.433	0.113	0.248	0.11	0.067	SLC13A4
TIIGTSPSL	0.451	0.12	0.356	0.128	0.093	SLC13A4
TRFKTTRII	0.044	0.094	0.047	0.08	0.659	AGO2
VLAFLLDMV	0.674	0.083	0.042	0.084	0.039	DOPEY2
VSAVWALCY	0.079	0.077	0.87	0.061	0.17	ZFP42
WEIFSFGPQ	0.049	0.014	0.021	0.583	0.009	ROR1
WTGWVCCVF	0.144	0.257	0.49	0.114	0.041	CENPL
YCTGPCHTF	0.074	0.369	0.571	0.151	0.139	PPP2R3A
YSGGEKPYL	0.192	0.078	0.479	0.078	0.29	COPB2
YSRIPKQSI	0.052	0.062	0.833	0.061	0.196	SVIL
YVVTEAGEL	0.223	0.05	0.441	0.132	0.158	GSDMC
YWQGNLDRF	0.061	0.655	0.111	0.093	0.09	POLR3C

Table 1: Neoepitope prediction for TCGA-A2-A0T2. The table contains all peptide sequences of length 9 that were predicted by NetMHC to bind to at least one of the sample's HLAs. For each HLA, the binding affinity is listed in their respective columns. Affinity scores can be interpreted as 1-log50k(IC50). Yellow cells indicate weak binders, green cells strong binders. The last column contains the gene symbol of the antigen-providing gene in the tumor.

References

1. Ott, P. A. *et al.* An immunogenic personal neoantigen vaccine for patients with melanoma. *Nature* **547,** 217–221 (2017).
2. Sahin, U. *et al.* Personalized RNA mutanome vaccines mobilize poly-specific therapeutic immunity against cancer. *Nature* **547,** 222–226 (2017).
3. Schubert, B., la Garza, de, L., Mohr, C., Walzer, M. & Kohlbacher, O. ImmunoNodes – graphical development of complex immunoinformatics workflows. *BMC Bioinformatics* **18,** 242 (2017).
4. Szolek, A. *et al.* OptiType: precision HLA typing from next-generation sequencing data. *Bioinformatics (Oxford, England)* **30,** 3310–3316 (2014).
5. Grossman, R. L. *et al.* Toward a Shared Vision for Cancer Genomic Data. *https://doi.org/10.1056/NEJMp1607591* **375,** 1109–1112 (2016).
6. Andreatta, M. & Nielsen, M. Gapped sequence alignment using artificial neural networks: application to the MHC class I system. *Bioinformatics (Oxford, England)* **32,** 511–517 (2016).
