# Supplementary material for: ClinOmicsTrailbc: a visual analytics tool for breast cancer treatment stratification
Source: Bioinformatics. 2019 Apr 30;35(24):5171–81. doi: 10.1093/bioinformatics/btz302 (PMC6954665; doi:10.1093/bioinformatics/btz302)
Supplement: btz302_Supplementary_Data [file btz302_supplementary_data.zip › btz302-Suppl_data/Supplementary_Data_S1.pdf]

Feature / Tool	Adjuvant therapy required	Survival time prediction	On-label drugs assessment	Off-label drugs assessment	Immuno-therapy assessment	Pathological markers as input	Genomics data as input	Epigenomics data as input	Transcript-omics data as input	Interactive results provided
**Adjuvant! Online** [1]	✓	✓	(✓)	✗	✗	✓	✗	✗	✗	✗
**PREDICT** [2]	✓	✓	(✓)	✗	✗	✓	✗	✗	✗	✗
**CancerMath** [3]	(✓)	✓	(✓)	✗	✗	✓	✗	✗	✗	✗
**Oncotype DX** [4]	✓	✗	(✓)	✗	✗	✓	✗	✗	✓	✗
**MammaPrint** [5]	✓	✗	✗	✗	✗	✓	✗	✗	✓	✗
**DrugTargetInspector** [6]	✗	✗	✓	✓	✗	✗	✓	✗	✓	✓
**CeGaT** [7]	✗	✗	(✓)	✗	✓	✗	✓	✗	✗	✗
**FoundationOne CDx** [8]	✗	✗	✓	✓	✓	✗	✓	✗	✗	✗
**ClinOmicsTrail^{bc}** [9]	✗	✗	✓	✓	✓	✓	✓	✓	✓	✓

Table 1. Comparison of different tools for clinical breast cancer decision support. The tools are assessed in terms of their considered input data types, the performed analyses / predictions and the presentation of the results. The green checkmark indicates that a certain feature is given, the red cross means that the considered feature is not provided and parentheses stand for a very limited extend to which a feature is considered / provided.

References

[1] Ravdin, Peter M., et al. "Computer program to assist in making decisions about adjuvant therapy for women with early breast cancer." *Journal of clinical oncology* 19.4 (2001): 980-991.

[2] dos Reis, Francisco J. Candido, et al. "An updated PREDICT breast cancer prognostication and treatment benefit prediction model with independent validation." *Breast Cancer Research* 19.1 (2017): 58.

[3] Website. URL: http://www.lifemath.net/cancer/breastcancer/therapy/index.php, last accessed: 2018-08-01

[4] Paik, Soonmyung, et al. "A multigene assay to predict recurrence of tamoxifen-treated, node-negative breast cancer." *New England Journal of Medicine* 351.27 (2004): 2817-2826.

[5] Slodkowska, Elzbieta A., and Jeffrey S. Ross. "MammaPrint™ 70-gene signature: another milestone in personalized medical care for breast cancer patients." *Expert review of molecular diagnostics* 9.5 (2009): 417-422.

[6] Schneider, Lara, et al. "DrugTargetInspector: An assistance tool for patient treatment stratification." *International journal of cancer* 138.7 (2016): 1765-1776.

[7] CeGaT. URL: https://www.cegat.de

[8] FoundationOne CDx. URL: https://www.foundationmedicine.com/genomic-testing/foundation-one-cdx

[9] Manuscript under submission. URL: https://clinomicstrail.bioinf.uni-sb.de
