# Supplementary material for: ClinOmicsTrailbc: a visual analytics tool for breast cancer treatment stratification
Source: Bioinformatics. 2019 Apr 30;35(24):5171–81. doi: 10.1093/bioinformatics/btz302 (PMC6954665; doi:10.1093/bioinformatics/btz302)
Supplement: btz302_Supplementary_Data [file btz302_supplementary_data.zip › btz302-Suppl_data/Supplementary_Data_S2.pdf]

Implementation of the web service

ClinOmicsTrail^{bc} is implemented as a web service, based on a multi-layer architecture that ensures extensibility and maintainability. The user-friendly interface leads the user through the data upload and analysis steps and provides additional help and information along the way. The interactivity of the presented results allows covering several layers of granularity without compromising ease-of-use and interpretability. In order to further facilitate the interpretability of the results, we provide interactive explanations (e.g., on the meaning of table entries, scores or indicator symbols) within the result visualizations. All visualizations and (intermediate) results can be downloaded for further processing or reporting.

The user frontend is based on HTML5 and the JavaEE platform, in combination with the Thymeleaf template engine. JavaScript, JQuery, and AJAX are used for client–server communication with a RESTful API that allows to set up and run computationally intensive tasks. Results are visualized using the DataTables plug-in for JQuery and the JavaScript libraries of Chart.js, D3 and Highcharts. The underlying database content is regularly updated in a semi-automatized manner.

In order to account for data safety and privacy, we securely transmit data between the client and the server using HTTPS. In a password-protected area, input files and analysis results can be viewed and completely deleted at any time. Inactive sessions will be deleted after 30 days.

ClinOmicsTrail^{bc} can be freely accessed at https://clinomicstrail.bioinf.uni-sb.de and is optimized for Google Chrome, Mozilla Firefox, and Apple Safari. Registration is possible, but not required.
